# Visual mismatch negativity and stimulus-specific adaptation: the role of stimulus complexity

**DOI:** 10.1007/s00221-019-05494-2

**Published:** 2019-02-26

**Authors:** Petia Kojouharova, Domonkos File, István Sulykos, István Czigler

**Affiliations:** 1 Institute of Cognitive Neuroscience and Psychology, Research Centre for Natural Sciences, Hungarian Academy of Sciences, P.O. Box 286, Budapest, 1519 Hungary; 2 Doctoral School of Psychology, Eötvös Loránd University, Budapest, Hungary; 3 Institute of Psychology, Eötvös Loránd University, Budapest, Hungary

**Keywords:** Visual mismatch negativity, Automatic deviant detection, Stimulus-specific adaptation, Stimulus complexity

## Abstract

The present study investigated the function of the brain activity underlying the visual mismatch negativity (vMMN) event-related potential (ERP) component. Snowflake patterns (complex stimuli) were presented as deviants and oblique bar patterns (simple stimuli) as standards, and vice versa in a passive oddball paradigm. Control (equiprobable) sequences of either complex shape patterns or oblique bar patterns with various orientations were also presented. VMMN appeared as the difference between the ERP to the oddball deviant and the ERP to the control (deviant *minus* control ERP difference). Apart from the shorter latency of the vMMN to the oblique bar pattern as deviant, vMMN to both deviants was similar, i.e., there was no amplitude difference. We attributed the function of the brain processes underlying vMMN to the detection of the infrequent stimulus type (also represented in memory) instead of a call for further processing (a possibility for acquiring more precise representation) of the deviant. An unexpected larger adaptation (control *minus* standard ERP difference) to the snowflake pattern was also obtained. We suggest that this was due to the acquisition of a more elaborate memory representation of the more complex stimulus.

**Electronic supplementary material:**

The online version of this article (10.1007/s00221-019-05494-2) contains supplementary material, which is available to authorized users.

## Introduction

The detection and identification of the changes in the visual environment are essential tasks of the perceptual system. A part of the change detection mechanisms are the brain processes underlying an event-related potential (ERP) component within the 120–350 ms post-stimulus latency range termed the visual mismatch negativity (vMMN). VMMN is elicited by visual events that violate a regularity in a stimulus sequence, even if the eliciting stimuli are unrelated to the ongoing behavior. In experimental context, vMMN is usually investigated in the passive oddball paradigm. In most cases the participants perform a visual task, while visual events are presented simultaneously outside its context as unattended stimuli. The unattended events form a stimulus sequence that sets up some sort of regularity: The stimuli could be identical, come from the same category, or follow a more complex sequential regularity. The stimuli conforming to the regularity are the standard stimuli. The sequence is infrequently interspersed with deviant stimuli that may differ by a visual feature (color, orientation, direction of movement, shape, spatial frequency, etc.), belong to a different category (e.g., a different facial emotion), or violate the sequential regularity (e.g., an irregular repetition within a sequence of alternating stimuli) (for reviews see Kimura et al. 2011; Kremláček et al. 2016; Stefanics et al. 2014).

VMMN is a counterpart to the more frequently investigated auditory mismatch negativity (MMN) (for a review on auditory MMN see Näätänen et al. 2011), so it is not surprising that the same theoretical explanations are applied as to the function of the underlying brain processes. At present there are two accounts concerning the elicitation of both (auditory) MMN and vMMN: The *trace mismatch theory* (see Näätänen 1992; for a critical overview see; Winkler 2007) and the *predictive coding theory* (e.g., Friston 2010). The aim of the present study is to investigate the function(s) of the brain activity underlying vMMN by presenting standard and deviant stimuli that are highly different both in visual complexity and categorically in a passive oddball paradigm. In this case, the two accounts make different predictions about the emergence of vMMN.

According to the *trace mismatch theory*, the sensory memory representation (trace) of the standard is compared to the representation of the incoming event. In the case of a difference (mismatch), the emerging brain activity is a correlate of a call for further processing of the deviant event (the possibility that an event is processed at a deeper level, i.e., more precise processing of orientation, or in the case of meaningful stimuli, the meaning *per se*, and/or the specificity of the event within the category). This account is related to the orientation theory proposed by Sokolov (1963), together with the notion that due to the continuous task-related processing and/or due to the deviancy not being sufficiently salient, other aspects of the orienting reaction (changes in the autonomic nervous system activity, motor processes) are absent (for a detailed explanation see Näätänen 1992). However, it was demonstrated that the violation of complex regularities [e.g., short tones are followed by low tones, and long tones are followed by high tones (Paavilainen et al. 2007)] also elicits MMN without being limited to a specific parameter of the standard stimulus, therefore MMN was considered to be an index of detected violation of any registered regularities within the stimulus sequence (e.g., Winkler 2007).

The *predictive coding theory*, a more recent account regarding the underlying mechanism of both (auditory) MMN and vMMN, proposes that to minimize the use of energy resources, “biological systems should continually minimize their surprise about sensory states” (Auksztulewicz and Friston 2016, p 126). According to this account, mismatch components are error signals. The function of the processes underlying both (auditory) MMN and vMMN is the adjustment of the representation of incoming stimulation to the representation of the model of the recent environment, i.e., a cascade of processes until the error is eliminated (for reviews see Garrido et al. 2009; Stefanics et al. 2015). In the auditory modality a large body of research supports the predictive coding view (for a review see Bendixen 2014). In contrast, the evidence for a similar interpretation of vMMN is based on a small set of studies. These studies investigated the ERP effects of violated sequential regularities with no specific parameter attached to the standard and the deviant. Such studies include the deviant repetition of a stimulus in a sequence of alternating stimuli pairs (Czigler et al. 2006), the deviant repetition of emotion in a sequence of alternating emotions (Kimura et al. 2011), the second (deviant) member of pairs of dots with different colors among pairs of dots with the same color (Stefanics et al. 2011), the second standard after a deviant (Kimura et al. 2010), and the violation of a rotating rule (Kimura and Takeda 2015), all of which elicited a posterior negativity (vMMN).

Thus, there are two different assumptions about the function of the brain activity underlying (auditory) MMN and vMMN. According to the *trace mismatch theory*, vMMN is the result of an automatic and putatively partial identification of the deviancy, and the role of the processes underlying this ERP component is to promote a more elaborate processing of the eliciting event. The *predictive coding account*, on the other hand, hypothesizes another locus within the stream of information processing—vMMN is the correlate of elementary identification of the incoming events not just as a deviancy, but also what deviancy within the environmental model.

In the present study we presented oddball sequences with simple stimuli (oblique bar patterns) and complex patterns (snowflake patterns). In one condition the oblique bar pattern was the standard and the snowflake pattern was the deviant, and in the other their roles were reversed. The choice of highly different stimuli was deliberate to prevent model adjustment, and thus make it possible to compare the two accounts. In other words, the emergence of vMMN in these sequences would be difficult to attribute to it being the correlate of the adjustment of an environmental model to the incoming activity (a putative cascade of processes leading to the matching of bottom-up and top-down processes) as supposed by the *predictive coding theory* as it is difficult to imagine that the presence of a deviant snowflake modifies the representation of an oblique bar or vice versa. However, if vMMN is an index of the mismatch between the representation of standard and that of the deviant at an initial stage without the involvement the adjustment process and the mismatch at subsequent stages of the cascade of processes, the putative mechanism underlying vMMN is fairly similar to that posited by the *trace mismatch* account. In addition, within an orientation-related theory such as the latter account one may expect that a more salient deviant elicits a more robust vMMN (defined here as a larger amplitude and/or a shorter latency).

VMMN is often calculated as the difference between the ERP responses to the deviant and the standard stimuli (deviant *minus* standard). However, in the oddball paradigm, the stimulus-specific adaptation (SSA, see below) difference between the negative exogenous ERP components (mainly the N1 component) to the frequent (standard) and rare (deviant) stimuli may contribute to the deviant *minus* standard difference potentials. A goal of our experimental design is to isolate the *genuine* mismatch response. Therefore, we included equiprobable control sequences. In the equiprobable control sequences the probability of the (randomly presented) various stimuli is identical to the probability of the oddball deviant. Consequently, in the control sequence no specific rule can be violated by any of the stimuli (Jacobsen and Schröger 2001; Schröger and Wolff 1996). Accordingly, the deviant *minus* control difference is free of SSA, i.e., it is a *genuine* mismatch response. When applied in the strictest sense, the additional stimuli in the equiprobable control sequence should be outside the range of the standard and deviant, beyond the critical deviant parameter. In certain cases (e.g., the pitch of auditory stimuli) the standard-deviant range can be defined reasonably. This is because the tonotopic representation in the auditory cortex corresponds to the spectrum of tone frequencies. However, there is no continuum between the oblique bar and the snowflake patterns, and we cannot apply the equiprobable control in a strict way. Here, we presented an equiprobable sequence with oblique bar patterns of various orientations, and another sequence with various complex shapes (for a review of vMMN studies using the equiprobable control procedure see File et al. 2017).

The investigation of SSA (decreased activity to stimulus repetition) is not the focus of the study, but it should be addressed. SSA is well-known at various levels of brain activity, behavioral effects, and subjective experiences, from single cell recording (Sawamura et al. 2006) to conscious experience (Clifford et al. 2010; Gibson 1937; Krekelberg et al. 2006) and has also been labeled as refractoriness or habituation (for a discussion see O’Shea (2015)). It is considered to be a consequence of memory acquisition or the result of memory update (e.g., Gonsalves et al. 2005; Grill-Spector et al. 2006; Lafontaine et al. 2016; Sayres and Grill-Spector 2006). Including equiprobable sequences in the design of the present study allows not only for isolating the *genuine* vMMN, but also for addressing the presence of SSA computed as the control *minus* standard difference (Kimura 2012), even if the paradigm is not optimal for investigating SSA. This part of the study was exploratory.

In addition to the posterior region where vMMN is expected to emerge, frontal and central areas were also included in the analysis with exploratory purposes as there is an indication that there is a vMMN generator in the frontal areas (e.g., Kimura et al. 2010).

To sum up, there are three predictions regarding vMMN. First, a mismatch mechanism whose function is the mere indication of the deviancy as suggested by the *trace mismatch theory* predicts a similar vMMN in the conditions with complex and simple deviant stimuli. Second, a more salient deviant is expected to elicit a larger vMMN if this component is related to a call for further processing, i.e., an orienting reaction is initiated. Last, the *predictive coding* view does not rule out the emergence of vMMN, but the cascade of model adjustment is highly improbable in the present design, so in this case vMMN is not expected to emerge as a long-lasting negativity, i.e., in the 200–300 ms range.

## Methods

### Participants

Nineteen students [12 females; mean age 22.7 years (SD = 2.39 years)] participated in the experiment for partial course credit or payment. All had normal or corrected to normal vision, and no one reported any neurological or psychiatric diseases. Five participants were left-handed. The study was approved by the United Ethical Review Committee for Research in Psychology (Hungary), and was carried out in accordance with the Declaration of Helsinki. Written informed consent was obtained from all participants.

### Stimuli and procedure

**Fig. 1:**
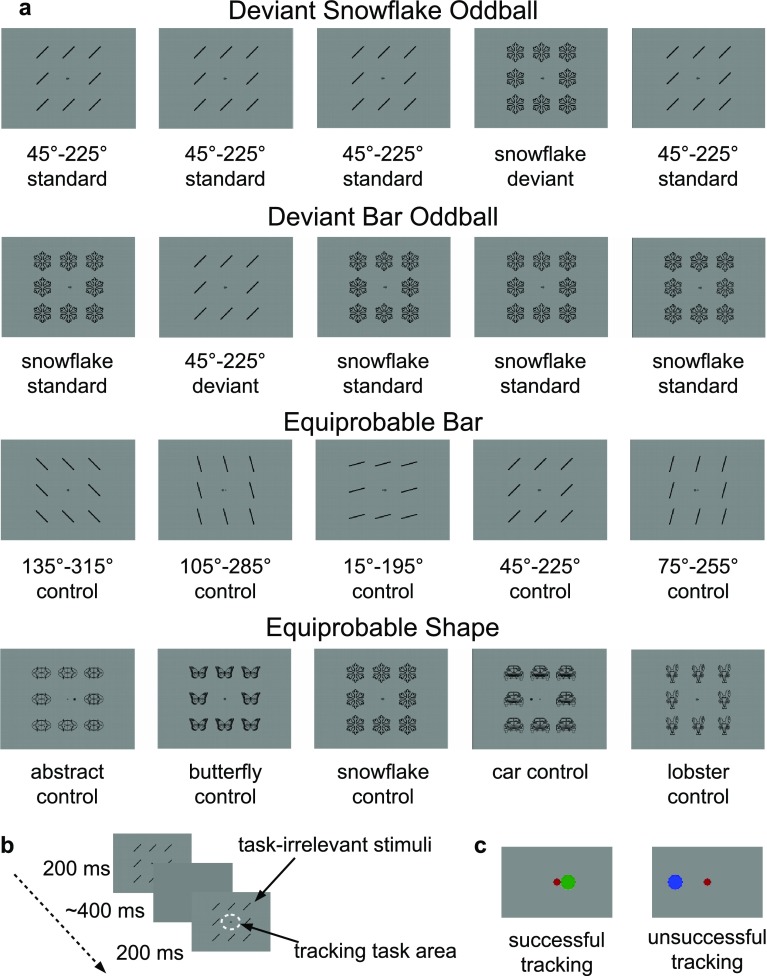
Experimental paradigm and stimuli. **a** Stimuli in each condition. **b** Experimental procedure. **c** The tracking task area in the case of successful and unsuccessful tracking

The experimental stimuli (summarized in Fig. 1 along with the experimental procedure) included both task-relevant and task-irrelevant stimuli presented on an 18″ CRT monitor (LG Flatron 915FT Plus, 75 Hz refresh rate, 1024 × 768 px screen resolution) at a viewing distance of 140 cm.

The purpose of the task-relevant stimuli (a tracking task) was to engage the participants’ attention. A red dot (RGB 0.6, 0, 0) with a diameter of 0.14° of visual angle served as a fixation point in the center of the screen. A green disc (0.29°, RGB 0, 0.5, 0) moved in a pseudorandom fashion around the fixation point. The participant’s task was to keep the green disc as close as possible to the fixation point by controlling its movement with the right and left arrow keys of a keyboard. Each time the distance between the green disc and the fixation point exceeded 0.73° in either direction, the color of the disc changed to blue (RGB 0, 0, 1) to provide visual feedback that the disc was in the “error zone”, and this was counted as an error. As long as the disc was in the “error zone”, it remained blue. Performance was measured with: (1) number of errors and (2) percentage of time the disc spent in the “error zone” during a sequence.

The purpose of the task-irrelevant stimuli was to elicit vMMN. Eight identical black oblique bars or line drawings (complex shapes) were arranged in a pattern (a 3 × 3 grid) around the tracking task. The vertical distance from the fixation point to a bar or a complex shape of the pattern was 2.88° of visual angle when it was situated above or below the fixation point, and the horizontal distance was 2.92° when it was on either side (see Fig. 1a). Each bar stimulus was 0.12° of visual angle wide and 1.74° long. Each complex shape was 2.19° of visual angle at its larger dimension. Stimuli were presented as dark objects (0.14 cd/m^2^, RGB 0, 0, 0) on a gray background (15.58 cd/m^2^, RGB 0.5, 0.5, 0.5).

The experiment included the following four conditions: an oddball condition with an oblique bar pattern as the standard and a snowflake pattern as the deviant stimuli (Deviant Snowflake Oddball); an oddball condition with a snowflake pattern as the standard and an oblique bar pattern as the deviant stimuli (Deviant Bar Oddball); an equiprobable condition with oblique bar patterns of five different orientations as control stimuli (Equiprobable Bar); an equiprobable condition with five different shape patterns as control stimuli (Equiprobable Shape). In the oddball conditions the bars had a 45°–225° orientation. In the Equiprobable Bar condition bars with orientations of 15°–195°, 75°–255°, 105°–285° and 135°–315° were added (i.e., the orientations were equally distributed within the 360°). In the Equiprobable Shape condition the additional shapes were a butterfly, a bilateral abstract pattern, the front view of a car, and a lobster. The only criterion for choosing the complex shapes was there being no obvious systematic difference between them.

Each condition had a total of 600 stimuli divided into three sequences of 200 stimuli for a total of 12 sequences. For the oddball conditions the probability was 0.8 for the standard and 0.2 for the deviant (480 standard and 120 deviant stimuli in each condition, 160 standard and 40 deviant stimuli in each sequence). In the equiprobable conditions each stimulus had a 0.2 probability, matching the deviant’s probability from the oddball conditions (120 stimuli of each overall, 40 of each within a sequence). The presentation order of the sequences was randomized for each participant.

The stimulus presentation time was 200 ms; the average inter-stimulus-interval (ISI) was 400 ms (range 360–440 ms in 13.33 ms steps). In the oddball conditions there were between 2 and 6 standard stimuli between two deviants, whereas in the equiprobable conditions the stimuli were randomized with no two identical stimuli occurring in succession.

The participant sat in a dark room in a comfortable chair with a keyboard on his/her lap in front of the screen on which the stimuli were presented. The experiment started with a 3-min practice during which only the tracking task was presented, and the participant was instructed to keep the green disc as close to the fixation point as possible. EEG was not recorded for the practice.

The experiment started after the practice. The participant was told to focus on the tracking task and ignore the distracting stimuli. After each sequence there was a short break, and the participant started the next sequence with a key press. Each sequence lasted for approximately 2 min. The number of errors was displayed on the screen after each sequence. The duration of the experimental part of the session was approximately 1 h.

A summary of the conditions and the stimuli can be found in Table 1a.

**Table 1:** **a** Conditions and stimuli.** b** Calculations for the difference waves (vMMN, SSA, vMMN *and* SSA).** c** Regions included in the analysis, each region was analyzed separately. d Factors of the univariate analysis of variance

(a) Conditions and stimuli			
Condition	Deviant stimulus	Standard stimulus	Control stimuli
Deviant bar oddball	Oblique bar with a 45°–225° orientation	Snowflake	–
Deviant snowflake oddball	Snowflake	Oblique bar with a 45°–225° orientation	–
Equiprobable bar	–	–	Oblique bars with orientations 45°–225°, 15°–195°, 75°–255°, 105°–285°,135°–315°
Equiprobable shape	–	–	Snowflake, butterfly, abstract pattern, car (front view), lobster

(b) Calculations for the difference waves	
vMMN	Deviant *minus* control = deviant stimulus (from an oddball condition) *minus* control stimulus (from the respective equiprobable condition)
SSA	Control *minus* standard = control stimulus (from the respective equiprobable condition) *minus* standard stimulus (from an oddball condition, last standard before a deviant only)
A combination of vMMN and SSA (for illustration purposes only)	Deviant *minus* standard = deviant stimulus (from one oddball condition) *minus* standard stimulus (from the other oddball condition, with the same physical properties, last standard before a deviant only)

(c) Regions included in the analysis		
Posterior region	Centroparietal region	Frontocentral region
PO3, POz, PO4, O1, Oz, O2	C1, Cz, C2, CP1, CPz, CP2	F1, Fz, F2, FC1, FCz, FC2

(d) Factors of the univariate analysis of variance	
Deviant *minus* control ANOVA factors	Stimulus type: oblique bar as deviant, snowflake as deviant
	Location: (PO3, O1 vs. POz, Oz vs. PO4, O2) or (F1, FC1 vs. Fz, FCz vs. F2, FC2)
	Anteriority: (PO3, POz, PO2 vs. O1, Oz, O2) or (F1, Fz, F2 vs. FC1 FCz, FC2)
Control *minus* standard ANOVA factors	Stimulus type: oblique bar as standard, snowflake as standard
	Location: (PO3, O1 vs. POz, Oz vs. PO4, O2) or (F1, FC1 vs. Fz, FCz vs. F2, FC2)
	Anteriority: (PO3, POz, PO2 vs. O1, Oz, O2) or (F1, Fz, F2 vs. FC1 FCz, FC2)

This experiment was realized using Cogent 2000 [within MATLAB (MathWorks, Inc. 2015)] developed by the Cogent 2000 team [Wellcome Department of Imaging Neuroscience (Cogent, http://www.vislab.ucl.ac.uk/Cogent/)].

### Recording and measuring electric brain activity

Brain electric activity was recorded [bandwidth: DC-100 Hz; sampling rate 500 Hz; Synamps2 amplifier, NeuroScan recording system (Compumedics Ltd)] with Ag/AgCl electrodes placed at 61 locations according to the extended 10–20 system using an elastic electrode cap (EasyCap, Brain Products GmbH). The reference electrode was on the nose tip, and the data were offline re-referenced to the average activity. Eye movements were recorded with four electrodes placed around the eyes. Horizontal EOG was recorded with a bipolar configuration between electrodes positioned lateral to the outer canthi of the eyes (one electrode on each side). Vertical eye movement was monitored with a bipolar montage between two electrodes, one placed above and one below the left eye. The impedance of all electrodes was kept below 10 kΩ. The EEG signal was band-pass-filtered offline with a non-causal Kaiser-windowed FIR filter (lowpass filter parameters: cutoff frequency of 30 Hz, beta of 12.2653, a transition band of 10 Hz; highpass filter parameters: cutoff frequency of 0.1 Hz, beta of 5.6533, a transition band of 0.2 Hz). Epochs with a duration of 600 ms, including a 100 ms pre-stimulus interval, were extracted for each event and averaged for each type of stimuli (see Data Analysis). The mean voltage during the 100 ms pre-stimulus interval served as the baseline for amplitude measurements, and epochs with an amplitude change exceeding 100 µV on any channel were excluded from further analysis.

### Data analysis

Performance on the tracking task was compared across conditions to ensure that participants attended to the task equally. The number of errors and percentage of time spent in the “error zone” were recorded for each sequence, averaged per condition for each participant, and then compared with a one-way non-parametric analysis of variance test (Friedman test) with the factor Condition (four levels: Deviant Snowflake Oddball, Deviant Bar Oddball, Equiprobable Bar, Equiprobable Shape).

Epochs were averaged for the following types of stimuli: (1) deviant for each oddball condition, (2) last standard immediately before a deviant for each oddball condition, and (3) the respective control stimuli (the 45°–225° bar and the snowflake) from each equiprobable condition. The number of averaged epochs for each of the six stimulus types was between 111 and 113 per participant; 5.70–6.89% of the epochs were rejected.

The analyses focused primarily on ERPs measured at the electrode sites over the posterior areas (PO3, POz, PO4, O1, Oz, and O2) within the 120–350 ms window, i.e., the expected locations and time window of vMNN as our main goal was to identify the presence of vMMN for stimuli from different categories. Because it was possible that the categorical difference also resulted in a difference elsewhere, two additional regions were also examined: a centroparietal (CP1, CPz, CP2, C1, Cz, C2) and a frontocentral (FC1, FCz, FC2, F1, Fz, F2) region. The choice of both additional regions was based on visual inspection of the ERPs and the scalp distributions to the difference waves.

To identify changes related to vMMN, the ERPs elicited by the control stimuli with the same physical properties in the equiprobable conditions were subtracted from the ERPs to the deviant stimuli (deviant *minus* control) (see Kimura et al. 2010). To assess the involvement of SSA, the ERP difference of the standard stimuli and the control stimuli sharing the same physical properties was also calculated (control *minus* standard). To illustrate the difference between the vMMN and the vMMN *plus* SSA, we present the deviant *minus* standard difference. This difference was calculated from the standard and deviant stimuli with identical physical properties, e.g., the ERP to the standard stimuli from the Deviant Bar Oddball condition was subtracted from the ERP to the deviant stimuli from the Deviant Snowflake Oddball condition (both were snowflake patterns).

One-sample *t* tests were run on the difference potentials to identify consecutive significant deviations from zero in the negative direction. Only significant deviations for at least 11 consecutive data points (20 ms) at all six electrode sites in a region were considered to indicate the presence of vMMN in the relevant condition for the deviant *minus* control difference potential (cf. Guthrie and Buchwald 1991). Peak and onset latencies were measured to assess the time of detecting the deviancy. Peak latencies were measured with a sliding window algorithm. As a first step, the algorithm finds the local maximum of the ERP. Then the algorithm examines whether it is a peak (i.e., the value is larger than both flanking values within the window) or a maximum (i.e., the value is the largest within the window, but it is also at the edge of the window). In the latter case, the window slides toward the maximum with the size of a half-window. The size of the sliding window was ten data points. The onset latency of the difference potentials was defined as the latency of the midpoint between the baseline and the peak amplitude and was calculated from the grand average with the jackknife-based method described by Miller et al. (1998), see also Luck (2005); Ulrich and Miller (2001). Amplitudes and integrated activity were measured to assess the magnitude of vMMN. Amplitude values were calculated as the average of the ± 10 ms range (a 20 ms duration) around the largest negativity in the grand average. Finally, the integrated activity (i.e., the magnitude of the activity as whole) was calculated as the sum of the amplitude values within the range for which the point-by-point differences between the deviant and control stimuli were statistically significant. In all cases, three-way univariate ANOVAs were calculated for each region with factors Stimulus Type (oblique bar pattern as deviant, snowflake pattern as deviant), Location (left, center, right), and Anteriority (posterior, anterior). Similar calculations were carried out for the control *minus* standard differences to determine the magnitude of SSA. When necessary, Greenhouse–Geisser corrections were used. For post hoc comparison the Tukey HSD test was applied. Only the main effects and interactions for Stimulus Type are reported in the text, but a summary of all results can be found in Supplementary Information 1. A summary of the performed calculations can be found in Table 1b–d.

The EEG data were processed with MATLAB R2015a (MathWorks, Inc. 2015) and the EEGLAB 13.6.5b toolbox (Delorme and Makeig 2004). Statistical analyses were performed with Statistica v13 (Statsoft, Inc. 2016).

## Results

### Behavioral results

The mean number of errors was: 0.75 (SD = 0.88) for the Deviant Snowflake Oddball condition, 1.0 (SD = 1.08) for the Deviant Bar Oddball condition, 0.93 (SD = 0.86) for the Equiprobable Bar condition, and 0.54 (SD = 0.49) for the Equiprobable Shape condition, i.e., the green disc went to the “error zone” less than once per sequence on average. There was no significant difference between the conditions (Friedman test, *χ*^2^(3, *N* = 19) = 3.8, *p* = 0.284). The mean percentage of time spent in the “error zone” was: 0.16% (SD = 0.26%) for the Deviant Snowflake Oddball, 0.17% (SD = 0.24%) for the Deviant Bar Oddball, 0.19% (SD = 0.24%) for the Equiprobable Bar, and 0.08% (SD = 0.07%) for the Equiprobable Shape. There was no significant difference between the conditions (Friedman test, χ^2^(3, *N* = 19) = 1.88, *p* = 0.597). This high performance^1^ suggests that participants attended to the task.

### Event-related potentials

**Fig. 2:**
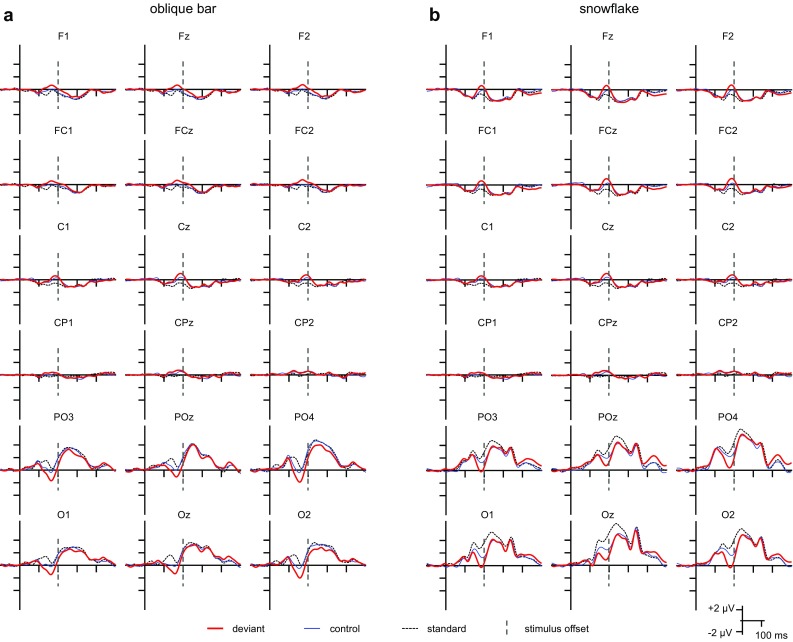
ERPs of the standard, deviant, and control stimuli for the oblique bar pattern (**a**) and the snowflake pattern (**b**) registered at posterior channels, centroparietal and frontocentral sites

As Fig. 2 shows, the ERPs to the oblique bar and the snowflake patterns were dominated by the P2 component within the 200–300 ms range at the posterior sites. A discernable N1 can be observed only for the oblique bar pattern as deviant. As a marked difference between the ERPs, only the snowflake pattern elicited offset components [a negative–positive response in the 100–200 ms range after stimulus offset (offset was at 200 ms after onset)]. The patterns were reversed, and the ERPs were smaller at the frontocentral sites. The morphology of the ERPs in the equiprobable sequences was similar to the respective ERPs from the oddball sequences, and visual inspection shows them to be between the ERPs to the respective deviant and standard stimuli in the vicinity of N1. The ERPs to the stimuli in the equiprobable conditions are depicted in Supplementary Information 2.

**Fig. 3:**
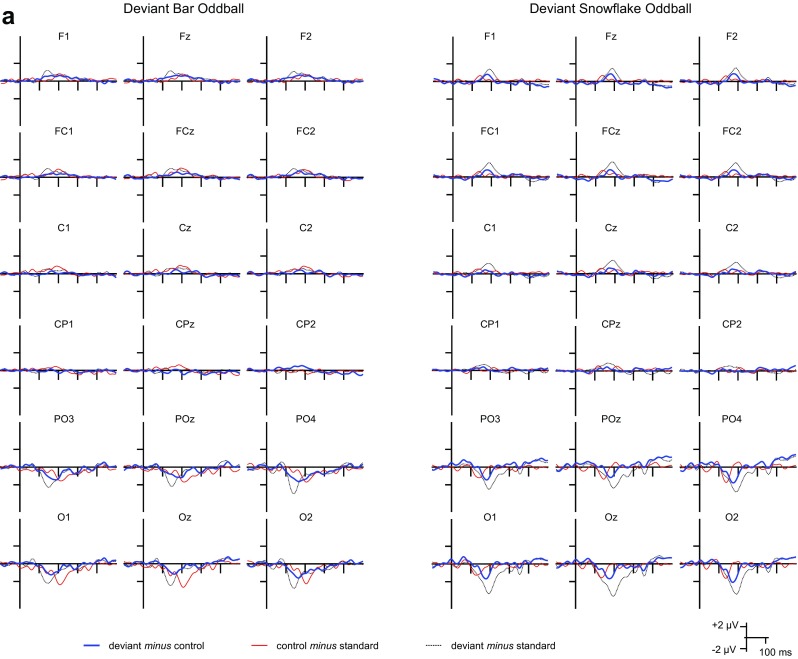

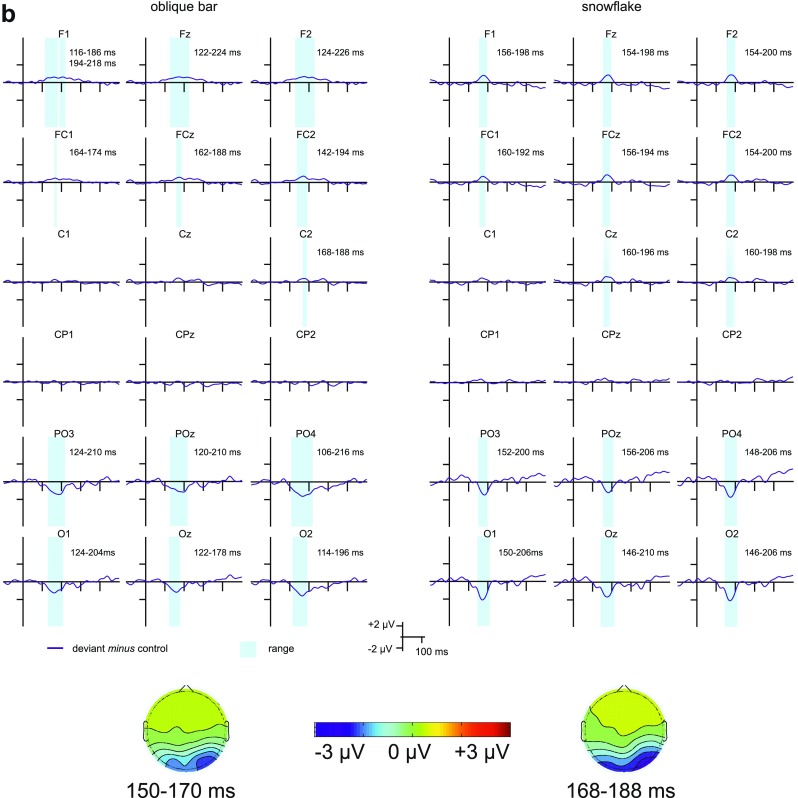

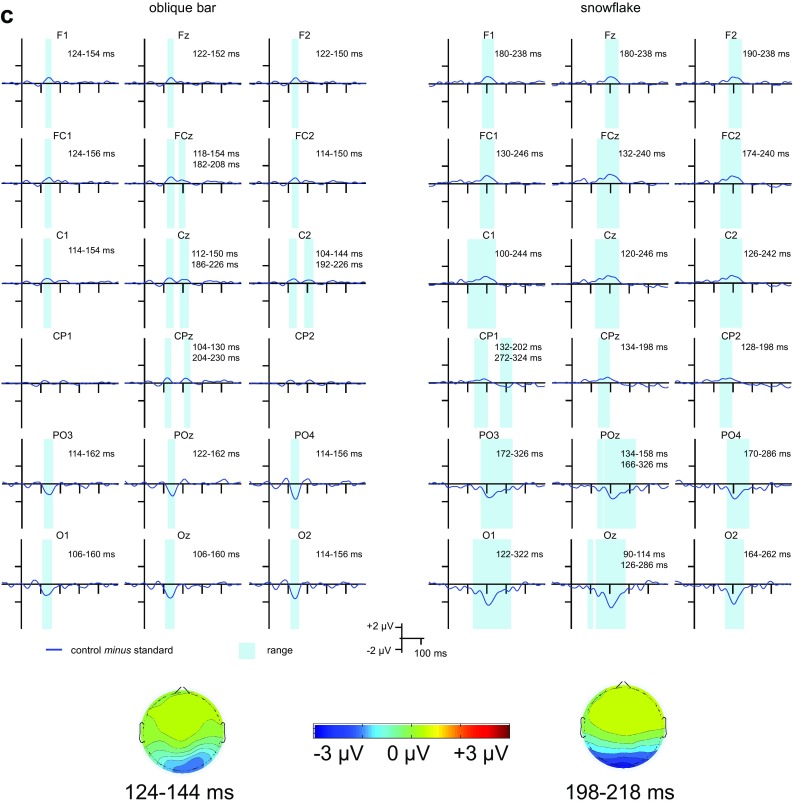
**a** The deviant *minus* control, control *minus* standard, and deviant *minus* standard difference potentials for the two oddball conditions registered at posterior, centroparietal and frontocentral sites. **b** The deviant *minus* control difference potentials for the oblique bar pattern and the snowflake pattern as deviant stimuli registered at posterior, centroparietal and frontocentral sites the ranges for the integrated activity as well as scalp distributions for the 20 ms range around the peak latency in the grand average at Oz. **c** The control *minus* standard difference potentials for the oblique bar pattern and the snowflake pattern as standard stimuli registered at posterior, centroparietal and frontocentral sites the ranges for the integrated activity as well as scalp distributions for the 20 ms range around the peak latency in the grand average at Oz

The deviant *minus* standard,^2^ deviant *minus* control and control *minus* standard difference potentials are depicted in Fig. 3a–c show the ranges for the integrated activity (the duration of consecutive data points for which the difference significantly deviated from 0 in the negative direction) around the peak latency in the grand average for the deviant *minus* control and control *minus* deviant difference potentials, respectively. Scalp distributions for the average of the 20 ms range around the peak latency in the grand average at Oz for each difference are also depicted.

According to the criterion [11 consecutive data points (20 ms) of significant deviation from 0], both the deviant oblique bars and the deviant snowflakes elicited vMMN at the posterior and frontocentral, but not at the centroparietal locations (Fig. 3b). Thus, our analysis was limited to those two regions. The narrowest range for the oblique bar pattern was 48 ms at PO3 for the posterior region and 32 ms at FC1 for the frontocentral region, and for the snowflake pattern it was 56 ms at Oz for the posterior region and 26 ms at FCz for the frontocentral region.^3^

Table 2 shows the average peak and onset latency values of the deviant *minus* control and the control *minus* standard difference potentials, the average peak amplitude values of these difference potentials, and the averaged integrated activity for the highlighted ranges.

**Table 2:** Average peak latencies, onset latencies, average peak amplitude values, and average integrated activity of the deviant *minus* control (vMMN) and control *minus* standard (SSA) difference potentials measured at the posterior (O1, Oz, O2, PO3, POz, PO4) and the frontocentral (FC1, FCz, FC2, F1, Fz, F2) regions for the oddball conditions (*S.E.M*. in parenthesis)

	Deviant *minus* control						Control *minus* standard					
	O1	Oz	O2	PO3	POz	PO4	O1	Oz	O2	PO3	POz	PO4
Peak latency (ms)												
Oblique bar	162.9 (6.64)	158 (5.20)	163. 8 (6.29)	168.8 (5.36)	161.5 (6.24)	162.9 (7.5)	133.5 (4.47)	136.8 (4.55)	135.7 (3.09)	137.1 (4.32)	140.2 (4.06)	132 (3.39)
Snowflake	173.4 (4.43)	177.4 (3.66)	175.9 (3.15)	181.6 (3.57)	180.8 (4.17)	176.9 (2.81)	211.5 (7.27)	207.9 (8.26)	202.7 (2.93)	241.4 (8.81)	249.0 (9.05)	211.7 (5.5)
Onset latency (ms)												
Oblique bar	126.7 (0.41)	124.4 (0.62)	123.4 (0.88)	130.6 (0.53)	119 (1.91)	114.1 (0.79)	104.5 (0.32)	107.9 (0.36)	113.7 (0.24)	113.6 (0.34)	123.2 (0.36)	117.2 (0.19)
Snowflake	151 (0.28)	147.2 (0.32)	148 (0.24)	156.7 (0.23)	157.8 (0.3)	148.6 (0.25)	177.8 (0.25)	176.1 (0.22)	177.5 (0.24)	182.7 (0.21)	182.9 (0.22)	178.7 (0.28)
Amplitude (µV)												
Oblique bar	− 1.22 (0.3)	− 1.14 (0.33)	− 1.55 (0.38)	− 1.42 (0.38)	− 1.17 (0.49)	− 1.6 (0.41)	− 1.23 (0.25)	− 1.51 (0.24)	− 1.56 (0.35)	− 1.18 (0.25)	− 1.28 (0.26)	− 1.65 (0.34)
Snowflake	− 1.62 (0.29)	− 1.64 (0.34)	− 2.03 (0.37)	− 1.39 (0.28)	− 1.13 (0.32)	− 1.61 (0.33)	− 2.31 (0.33)	− 2.59 (0.39)	− 2.17 (0.34)	− 1.53 (0.29)	− 1.66 (0.32)	− 1.65 (0.32)
Integrated activity (µV)												
Oblique bar	40.92 (9.79)	27.86 (6.36)	40.92 (9.79)	51.66 (11.87)	44.98 (13.21)	72.8 (16.4)	30.23 (5.91)	34.92 (5.92)	29.31 (6.93)	25.91 (5.09)	23.16 (4.47)	30.24 (6.6)
Snowflake	38.1 (7.57)	42.83 (9.52)	49.29 (10)	28.84 (6.72)	24.32 (7.85)	40.77 (9.29)	121.32 (19.82)	123.85 (21.42)	68.52 (11.18)	75.44 (11.57)	83.27 (14.44)	62.14 (11.16)

	Deviant *minus* control						Control *minus* standard					
	FC1	FCz	FC2	F1	Fz	F2	FC1	FCz	FC2	F1	Fz	F2
Peak latency (ms)												
Oblique bar	158.7 (8.2)	171.5 (5.76)	167.9 (5.08)	177.9 (6.94)	184 (7.44)	183.7 (7.0)	136.5 (3.31)	133.4 (3.91)	131.1 (4.06)	136.4 (3.84)	134.8 (4.23)	133.9 (3.66)
Snowflake	173.7 (3.75)	180.4 (3.76)	180.7 (5.28)	179.6 (3.39)	181.6 (3.29)	182 (3.51)	198.6 (4.85)	191.5 (6.42)	193.9 (5.51)	203.3 (3.84)	206.1 (3.9)	204.8 (4.34)
Onset latency (ms)												
Oblique bar	120 (0.89)	142.4 (2.28)	136.6 (1.24)	113.7 (0.58)	105.3 (2.72)	101.2 (3.14)	117.5 (0.32)	115.5 (0.32)	114.1 (0.37)	120.6 (0.4)	118.9 (0.29)	118.7 (0.29)
Snowflake	153.6 (0.33)	153.6 (0.21)	152.7 (0.24)	153.4 (0.34)	154.2 (0.3)	153.9 (0.23)	172.3 (0.41)	159.3 (2.91)	160.7 (2.94)	177.7 (0.39)	175.8 (0.43)	174.6 (0.35)
Amplitude (µV)												
Oblique bar	0.46 (0.2)	0.56 (0.21)	0.68 (0.18)	0.6 (0.26)	0.63 (0.23)	0.66 (0.2)	0.61 (0.16)	0.71 (0.19)	0.58 (0.15)	0.62 (0.15)	0.63 (0.17)	0.59 (0.16)
Snowflake	0.62 (0.19)	0.78 (0.24)	0.78 (0.21)	0.77 (0.18)	0.82 (0.19)	0.85 (0.19)	0.9 (0.19)	1 (0.22)	0.86 (0.18)	0.8 (0.2)	0.78 (0.21)	0.74 (0.18)
Integrated activity (µV)												
Oblique bar	3.29 (1.38)	7.71 (2.81)	15.76 (4.26)	28.72 (8)	28.72 (8)	29.31 (8.22)	9.6 (2.64)	12.1 (3.41)	9.6 (2.62)	9.22 (2.32)	9.37 (2.59)	8.28 (2.33)
Snowflake	9.77 (3.2)	14.21 (4.51)	16.52 (4.86)	14.96 (3.97)	16.33 (4.18)	17.7 (4.31)	27.03 (6.37)	42.72 (8.44)	34.13 (7.33)	20.2 (5.14)	19.97 (5.53)	20.6 (5.03)

According to a three-way univariate ANOVA of the peak latencies of the deviant *minus* control difference potentials for the posterior region with factors of Stimulus Type (oblique bar pattern as deviant, snowflake pattern as deviant), Location (PO3 and O1, POz and Oz, PO4 and O2), and Anteriority (PO4, POz, PO3 vs. O1, Oz, O2), the main effect of Stimulus Type was significant, *F*(1, 18) = 9.16, *p* = 0.007, η_p_^2^ = 0.337), and the latency of vMMN was longer for the deviant snowflake pattern. In an ANOVA with the same factors for the onset latencies, the vMMN latency remained longer for the snowflake pattern as deviant, but the difference did not reach significance, *F*(1, 18) = 4.06, *p* = 0.059, *F* and *p* corrected according to the jackknife-based method. In the ANOVA on the peak amplitude values there was no significant main effect of Stimulus Type. Finally, as an additional measure for the robustness of vMMN, the integrated activity was compared with the same ANOVA and factors. The interaction between Stimulus Type and Anteriority was significant, *F*(1, 18) = 13.99, *p* = 0.002, η_p_^2^ = 0.437. Post hoc tests revealed that the vMMN activity was larger for the oblique bar at the PO sites (*p* = 0.019).

The same analyses were performed for the frontocentral region with factors of Stimulus Type (oblique bar pattern as deviant, snowflake pattern as deviant), Location (FC1 and F1, FCz and Fz, FC2 and F2), and Anteriority (FC1, FCz, FC2 vs. F1, Fz, F2). For peak latencies there was the interaction between Anteriority and Stimulus Type, *F*(1, 18) = 5.69, *p* = 0.028, η_p_^2^ = 0.240. The latency for the oblique bar pattern as deviant was shorter only at the F sites and was shorter than the latency for the same at the FC sites, *p*s between 0.003 and 0.025. There was no difference in onset latencies or peak amplitudes. Concerning the vMMN activity, the only significant interaction including Stimulus Type was the Anteriority × Stimulus Type interaction, *F*(1, 18) = 5.73, *p* = 0.028, η_p_^2^ = 0.242 with the activity being larger for the oblique bar pattern as deviant at the FC sites than at F sites for both deviants.

Stimulus-specific adaptation effects were assessed with the control *minus* standard difference potentials. SSA was clearly visible in the posterior and frontocentral regions with the narrowest range being 40 ms for the oblique bar pattern as standard at POz for the posterior region and 28 ms at F2 for the frontocentral region, and 98 ms at O2 in the posterior region and 58 ms at F1 and Fz in the frontocentral region for the snowflake pattern. The case for the centroparietal region is less straightforward. SSA is clearly present for the snowflake pattern as standard at both C and CP sites in the time window of 100–250 ms at the C sites and 130–200 ms at the CP sites, whereas there are two time windows at Cz, C2, and CPz at approximately 100–150 ms and 180–230 ms after stimulus onset, the first time window also being present for C1. As it is unclear which time window should be used and there are two electrode sites that do not fulfill the criterion of 11 consecutive data points, further statistical analysis was not conducted for this region.

For the posterior region in an univariate three-way ANOVA with the same factors as in the analysis for the deviant *minus* control difference, the main effect of Stimulus Type was significant, *F*(1, 18) = 238.69, *p* < 0.001, η_p_^2^ = 0.930. Additionally, the Anteriority × Stimulus Type interaction also reached significance, *F*(1, 18) = 12.30, *p* = 0.003, η_p_^2^ = 0.406. The latency of SSA for the snowflake pattern as standard was longer overall, and longer at PO than at O sites (all *p*s < 0.001). The three-way ANOVA for the onset latency confirmed the main effect of Stimulus Type, *F*(1, 18) = 121.23, *p* < 0.001, respectively (jackknife-based method (Miller et al. 1998; Ulrich and Miller 2001), *F* and *p* corrected accordingly). The onset latency was longer for the snowflake pattern as standard. The larger adaptation of the snowflake pattern as standard stimuli is reflected by the larger peak amplitude of the difference potential. According to the ANOVA, the main effect of Stimulus Type was significant, *F*(1,18) = 4.46, *p* = 0.048, η_p_^2^ = 0.199. The interaction of Anteriority and Stimulus Type was also significant, *F*(1, 18) = 10.95, *p* = 0.004, η_p_^2^ = 0.378. Post hoc tests showed that the amplitude of SSA for the snowflake pattern as standard was larger only at O sites (*p*s < 0.001). To illustrate the large adaptation difference, the integrated activities were also compared (see Table 2). All main effects and interactions including the factor Stimulus Type were significant: Stimulus Type, *F*(1, 18) = 29.80, *p* < 0.001, η_p_^2^ = 0.623, Anteriority × Stimulus Type, *F*(1, 18) = 12.90, *p* = 0.002, η_p_^2^ = 0.418, Stimulus Type × Site, *F*(2, 36) = 7.12, *p* = 0.003, η_p_^2^ = 0.283, and the triple interaction of Anteriority × Stimulus Type × Location, *F*(2, 36) = 4.62, *p* = 0.016, η_p_^2^ = 0.204. The SSA activity was larger overall for the snowflake pattern as standard, larger for the snowflake at O1, Oz, PO3 and POz, and larger at O1 and Oz than the PO sites (*p*s < 0.029).

The same analyses were performed for the frontocentral region. For peak latencies there was only a main effect of Stimulus Type, *F*(1, 18) = 166.92, *p* < 0.001, η_p_^2^ = 0.903 with the latency being longer for the snowflake pattern as standard. The result for the onset latency was the same, *F*(1, 18) = 6.57, *p* = 0.019. There was no difference in peak amplitude at the frontocentral sites; however, for the SSA integrated activity all main effects and interactions including the Stimulus Type with the exception of the Stimulus Type × Location interaction were significant: Stimulus Type, *F*(1, 18) = 10.76, *p* = 0.004, η_p_^2^ = 0.374, Anteriority × Stimulus Type, *F*(1, 18) = 7.25, *p* = 0.015, η_p_^2^ = 0.287, and the triple interaction of Anteriority × Stimulus Type × Location, *F*(2, 36) = 5.25, *p* = 0.010, η_p_^2^ = 0.226. The activity was overall larger for the snowflake pattern as standard, larger at Oz than O1 and O2, and at Oz and O2 than at POz and PO4.

All results from the statistical analyses can be found in Supplementary Information 1. An additional analysis of the time course of the adaptation of the standard is briefly summarized in Supplementary Information 3. An analysis of the differences in scalp distributions between conditions for the deviant *minus* control and the control *minus* standard differences based on the method described by Karniski, Blair, and Snider (1994) is briefly described in Supplementary Information 4.

## Discussion

For both the oblique bar pattern and the snowflake pattern vMMN emerged at the posterior region. Both the peak latency and the onset latency of vMMN were shorter for the oblique bar pattern as deviant compared to the snowflake pattern as deviant (refer to Table 2 for the magnitude of the difference), although for the latter difference only a tendency was observed. The peak vMMN amplitude was less than − 2 µV for both patterns, and the difference was not significant. The integrated activity did not differ between the two oddball conditions either. At the frontocentral electrode sites there was a smaller positivity in approximately the same range as the negativity in the posterior region. There were no differences in peak or onset latency between the two deviants as well as no overall difference in either peak amplitudes or integrated activity. The scalp distributions for the deviant *minus* control difference at the peak latencies did not differ (see Supplementary Information 4).

The main purpose of the study was to investigate three issues. First, according to the interpretation of the *trace mismatch explanation*, vMMN is no more than a sign of the detection of a deviant event. In this respect, the expectation is a similar vMMN to simple and complex deviants. The similarity in magnitude of the deviant *minus* control differences between the vMMN to the oblique bar pattern and that to the snowflake pattern stimuli corresponds to this interpretation. Second, if the more salient deviant elicits a more robust vMMN, such a result can be attributed to a call for further processing (and possibly to the further processing) of the more complex deviant. The results did not support this possibility. Third, according to the interpretation of *the predictive coding account*, vMMN is a correlate of a cascade of adjustment processes between the models of the environment and the representation of incoming stimulation. It is difficult to interpret the emergence of vMMN in the 200–300 ms range to deviants that are unrelated to the standard as a correlate of the adjustment processes. Importantly, we do not claim that the vMMN is not sensitive to such a sequence of processes in cases in which an adjustment between the incoming stimulation and the updating environmental models is a possibility. The present results only show that such a mechanism is not necessary for the emergence of vMMN.

As for the automatically acquired and implicit (non-conscious) memory, it claims that the function of this system is to maintain a representation of the regular characteristics of the environment, and the process underlying the mismatch components is to update the memory system (Winkler and Czigler 1998; Winkler and Schröger 2015). Here, the shorter vMMN latency to the oblique bar pattern (a simple stimulus) indicates that deviancy is detected earlier in the context of complex stimuli than a complex deviant event within the sequence of more simple ones. When the standard and the deviant are similar [e.g., belonging to the same category (oblique lines: Kimura et al. (2009, 2010); facial categories and emotions: Yu et al. (2017), Vogel et al. (2015), Kreegipuu et al. (2013); left vs. right hand: Stefanics and Czigler (2012)], deviant-related negativity included longer latency ranges, or there were mismatch components in various ranges, whereas in the case of highly different standard and deviant (e.g., symmetric vs. asymmetric patterns), the difference potential was confined to an earlier and narrower latency range. Furthermore, using similar standards and deviants such as disappearing parts of an object (Sulykos et al. 2017) and checkerboards with alternating locations of the dark and light squares (Sulykos et al. 2018), an identical earlier phase of the vMMN appeared for both younger and older groups, whereas the later part was absent or diminished in the elderly. This age-related difference can be interpreted as preserved detection of a deviance, but compromised identification of the deviancy.

VMMN for deviant oblique bars has been obtained in former studies using an equiprobable control with within-category stimuli (i.e., oblique bars with a different orientation) as standards, but the results are equivocal. Astikainen et al. (2008) and Kimura et al. (2009) presented single bars. Astikainen et al. (2008) obtained a deviant *minus* control difference in the 185–205 ms range. In the study by Kimura et al. (2009) applying an equiprobable control eliminated the difference in the range that corresponded to a posterior negativity (N1), but vMMN emerged at a later latency range (200–250 ms). The results of Kimura et al. (2010) were similar. Kimura and Takeda (2013) used oblique bar patterns as stimuli, similar to the present study, and as in their earlier studies, the control eliminated the early part of the response, but preserved the later difference. Finally, File et al. (2017) presented a texture consisting of oblique bars. In this study the control procedure fully eliminated the deviance effect. It seems that a complex interaction occurs between task-related attentional demand [auditory task: Astikainen et al. (2008), identification of a feature of the vMMN-related stimuli: Kimura et al. (2009, 2010); visual discrimination: Kimura and Takeda (2013); video games: File et al. (2017)] and the stimulus characteristics (single bar, bars around the task-field, texture of bars in non-attended part of the visual field). Early vMMN effects can be considered as a detection of change, and later effects as an identification of deviancy (Sulykos et al. 2017). This way the later effect may correspond to what is suggested by the predictive coding accounts (e.g., Stefanics et al. 2015). In the present study, however, the adjustment of the environmental model acquired by the standard and the incoming stimulus is highly improbable in either of the conditions.

Regarding the frontocentral region, we found that the pattern of the ERPs to the standard, deviant, and control stimuli as well as the difference waves is similar to the pattern at the posterior region but reversed and smaller. An additional analysis on peak and onset latencies with a four-level Anteriority factor (O1. Oz, O2 vs. PO3, POz, PO4 vs. FC1, FCz, FC2 vs. F1, Fz, F2) found a main effect of Anteriority for peak latencies, *F*(3, 54) = 4.85, *p* = 0.005, ε = 0.858, η_p_^2^ = 0.212, but only the latency at the F sites was somewhat longer (*p*s between 0.003 and 0.053 compared to the other regions), and there was no difference for onset latencies, *F*(3, 54) = 0.29, *p* = 0.834, ε = 0.525. Thus, the present data are not sufficient for distinguishing between a possible frontal source, a phase reversal, or use of the average reference.

As an unexpected finding, the SSA to the more complex snowflake pattern was larger than to the oblique bar pattern, which difference was observable for both peak amplitude and integrated activity at the posterior sites and for the integrated activity at the frontocentral sites. In addition, both the peak latency and the onset latency of the SSA were shorter in the case of the oblique bar pattern as standard at both regions. The scalp distributions for the control *minus* standard difference at the peak latencies differed, but the difference did not reach significance (Supplementary Information 4). For the oblique bar pattern as standard in the Deviant Snowflake Oddball condition, the ERP difference between the control and standard was confined to a narrow range. In this study, the stimuli did not elicit a detectable N1 component, but the lack of identified negativity in the N1 range does not exclude that superposition of various activities that cancelled each other in this range (Luck 2005). However, in the Deviant Bar Oddball condition (snowflake pattern as standard), the adaptation-related period included not only the N1, but also the P2 range. Therefore, it is difficult to argue that the standard-related effect is due to a simple refractoriness (or fatigue) of low-level input structures, reflected by the reduction of the N1 component. The long-lasting adaptation effect on the more complex stimulus argues against the possibility that the stronger adaptation was simply the consequence of an additive effect of low-level processes (e.g., the adaptation of various orientation-specific visual structures). It seems that in the case of complex stimuli SSA is a more powerful process than in the case of simple stimuli. Recently, Amado and Kovács (2016) reported data showing that adaptation/repetition suppression fully explained the ERP difference between the deviant and standard for complex stimuli such as faces and chairs. We suggest that the adaptation difference in the present study was due to the faster build-up of the representation of a simple regular event (oblique bar pattern), as reflected by the latency of the control *minus* standard difference potential. The robust adaptation for complex stimuli (the snowflake pattern as standard), on the other hand, is an indicator of the build-up of a more elaborated representation of the standard.

One limitation of the present study is that the traditional oddball paradigm is not particularly optimal for investigating stimulus-specific adaptation (SSA). In this respect, the reversal of the deviant-standard relationship within short sequences (roving standard paradigm) is a more promising method (e.g., Baldeweg et al. 2004). Furthermore, a comparison of the deviant-related effects in the oddball paradigm and in paradigms developed for investigating adaptation is a more direct possibility (Bodnar et al. 2017). Another limitation in the present design is that it is impossible to compare the precise level of adaptation in the two equiprobable conditions for the oblique line and the snowflake stimuli. This difference may contribute to the difference between the deviant *minus* standard difference potentials and the deviant *minus* control difference potentials in the oblique line vs. snowflake conditions.

## Conclusions

The visual mismatch negativity (vMMN), an event-related potential (ERP) difference between the deviant stimuli from oddball sequences and the corresponding identical stimuli from equiprobable sequences, was fairly similar for a simple deviant within a sequence of complex events and a complex deviant within a sequence of simple events. VMMN in the present design indicates that this activity does not require the adjustment of a predictive model and the incoming activity, and vMMN is not directly connected to orientation processes, i.e., it was not larger for the more salient stimulus. Stimulus-specific adaptation is stronger for complex stimuli. The adaptation difference is likely to indicate differences in the automatic acquisition of the representation of simple and more complex events.

## Electronic supplementary material

Below is the link to the electronic supplementary material.

Supplementary material 1. A summary of all results of the statistical analyses (univariate ANOVA) (PDF 405 KB)

Supplementary material 2. ERPs to the stimuli in the two equiprobable condition (PDF 371 KB)

Supplementary material 3. Adaptation of the standard stimuli in the Deviant Snowflake Oddball condition and in the Deviant Bar Oddball condition (PDF 398 KB)

Supplementary material 4. A comparison of topographic maps with the permutation method described by Karniski et al. (1994) (PDF 954 KB)

## Footnotes

1 There is no absolute measure of performance to which to compare the participants’ results in this task. Consequently, performance can only be compared between conditions. However, it is possible to obtain number of errors and time spent in the “error zone” for the length of an experimental sequence if the task is left to run without human interference. The task was run 96 times with the length of each run being equal to one experimental sequence (2 min), which averaged 11.45 errors and 53.39% of time spent in the “error zone” per sequence. This would be the performance if the participant does not do the task at all.

2 Deviant *minus* standard differences were calculated as the difference between the oblique bar pattern as a deviant minus the oblique bar as a standard, and the snowflake pattern as a deviant minus the snowflake pattern as a standard. These differences involve both deviant-related and stimulus-specific adaptation effects; therefore we present these differences only as an illustration.

3 The range at FC1 for the snowflake pattern was 10 ms; however, this site was included for the sake of the statistical analysis. For both conditions there is a monotonic linear increase from FC1 to FC2, although the slope for the oblique bar pattern is steeper than the slope for the snowflake pattern, and the small value at FC1 likely reflects the continuation of the linear trend as well as a distribution slightly to the right for vMMN at the FC electrode sites. The latter was also the reason for our decision to include the frontocentral region in the case of the snowflake pattern as a deviant, even though there was one electrode site (FC1) at which the range of consecutive data points significantly different from 0 did not reach 20 ms.
