# Supplementary material for: Visual mismatch negativity and stimulus-specific adaptation: the role of stimulus complexity
Source: Exp Brain Res. 2019 Feb 26;237(5):1179–94. doi: 10.1007/s00221-019-05494-2 (PMC6557884; doi:10.1007/s00221-019-05494-2)
Supplement: Supplementary file 1 — Supplementary material 1. A summary of all results of the statistical analyses (univariate ANOVA) (PDF 405 KB) [file 221_2019_5494_MOESM1_ESM.pdf]

Online Resource 1

Article title: Visual mismatch negativity and stimulus-specific adaptation: The role of stimulus complexity

Journal: Experimental Brain Research

Authors:

Petia Kojouharova, Institute of Cognitive Neuroscience and Psychology, Research Centre for Natural Sciences, Hungarian Academy of Sciences; Doctoral School of Psychology, Eötvös Loránd University. kojouharova.petia@ttk.mta.hu

Domonkos File, Doctoral School of Psychology, Eötvös Loránd University; Institute of Psychology, Eötvös Loránd University; Institute of Cognitive Neuroscience and Psychology, Research Centre for Natural Sciences, Hungarian Academy of Sciences

István Sulykos, Institute of Cognitive Neuroscience and Psychology, Research Centre for Natural Sciences, Hungarian Academy of Sciences.

István Czigler, Institute of Cognitive Neuroscience and Psychology, Research Centre for Natural Sciences, Hungarian Academy of Sciences.

Supplementary Information 1

Summary of all results of the three-way univariate ANOVA with factors of Stimulus Type (oblique bar pattern, snowflake pattern), Location (left (O1 and PO3 or FC1 and F1), center (Oz and POz or FCz and Fz), right (O2 and PO4 or FC2 and F2)), and Anteriority (anterior (PO3, POz, PO4 or F1, Fz, F2), posterior (O1, Oz, O2 or FC1, FCz, FC2)). If the result of the multivariate ANOVA (Wilk's Λ) was different, it was also reported. All figures show mean values and *S.E.M.* The results are summarized in Tables S1 through S16.

deviant *minus* control						
Posterior region (O1, Oz, O2, PO3, POz, PO4)						
Peak latencies						
Effects and interactions	*F*	df 1	df 2	*p*	ε	η_p^2
**Stimulus Type**	**9.16**	**1**	**18**	**0.007**	**-**	**0.337**
Anteriority	1.23	1	18	0.283	-	0.064
Location	0.45	2	36	0.612	0.831	0.027
Anteriority × Stimulus Type	0.05	1	18	0.826	-	0.003
Location × Stimulus Type	1.42	2	36	0.256	0.998	0.073
Anteriority × Location	1.14	2	36	0.332	0.982	0.059
Anteriority × Location × Stimulus Type	0.03	2	36	0.969	0.782	0.002

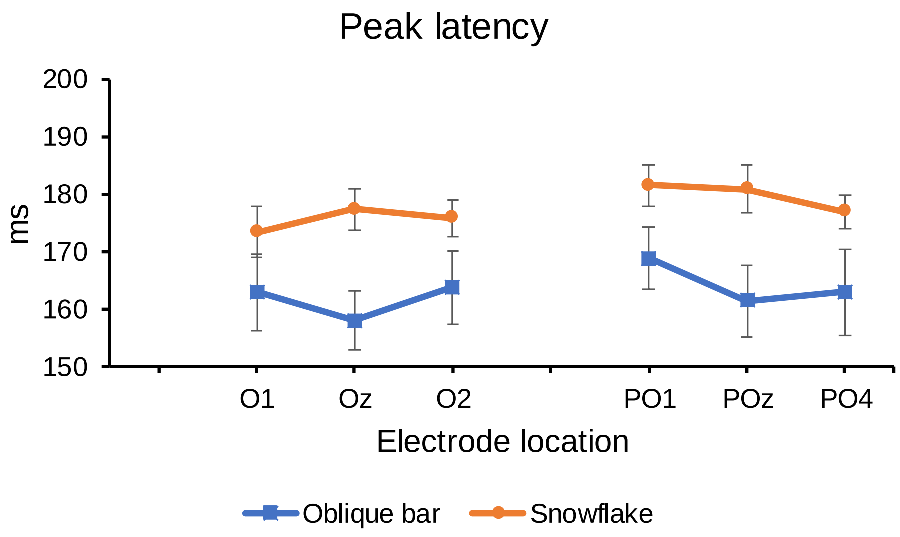

Table S1. Univariate ANOVA for the peak latencies at the posterior region for the deviant *minus* control difference wave. Factors: Stimulus Type (oblique bar pattern, snowflake pattern), Location (O1 and PO3, Oz and POz, O2 and PO4), Anteriority (O1, Oz, O2 vs. PO3, POz, PO4). Overall, the peak latency of the snowflake pattern as deviant was longer than that of the oblique bar pattern as deviant (p = 0.007)

deviant *minus* control						
Posterior region (O1, Oz, O2, PO3, POz, PO4)						
Onset latencies						
Effects and interactions	*F*	df 1	df 2	*p*	ε	η_p^2
Stimulus Type	4.06	1	18	0.059	-	-
Anteriority	0.03	1	18	0.86	-	-
Location	0.67	2	36	0.515	0.805	-
Anteriority × Stimulus Type	0.46	1	18	0.506	-	-
Location × Stimulus Type	0.1	2	36	0.909	0.843	-
Anteriority × Location	0.34	2	36	0.716	0.618	-
Anteriority × Location x Stimulus Type	0.18	2	36	0.837	0.632	-

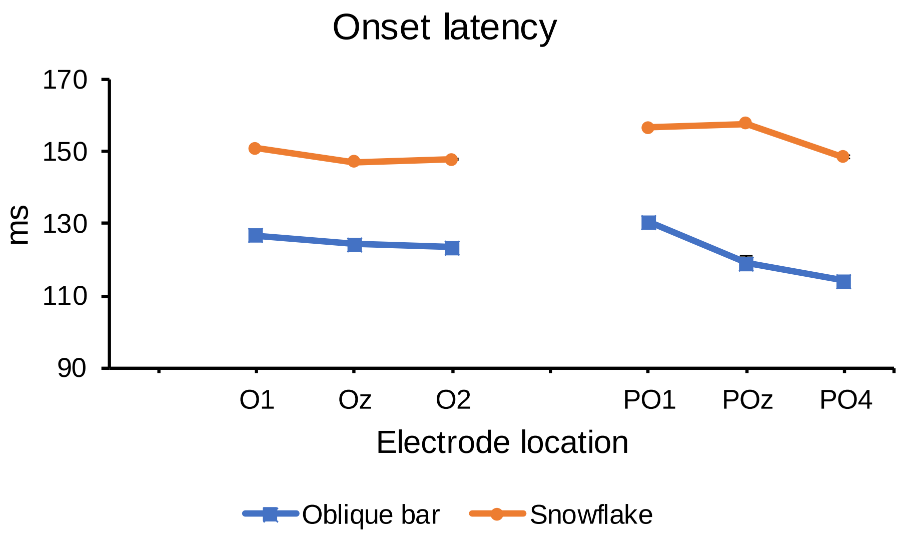

Table S2. Univariate ANOVA for the onset latencies at the posterior region for the deviant *minus* control difference wave. Factors: Stimulus Type (oblique bar pattern, snowflake pattern), Location (O1 and PO3, Oz and POz, O2 and PO4), Anteriority (O1, Oz, O2 vs. PO3, POz, PO4). The onset latency for the snowflake deviant was longer, but the difference did not reach significance (p = 0.059)

deviant *minus* control						
Posterior region (O1, Oz, O2, PO3, POz, PO4)						
Peak amplitudes						
Effects and interactions	*F*	df 1	df 2	*p*	ε	η_p^2
Stimulus Type	0.35	1	18	0.56	-	0.019
Anteriority	0.44	1	18	0.516	-	0.024
Location	3.31	1.44	25.96	0.066	0.721	0.156
Anteriority × Stimulus Type	3.75	1	18	0.069	-	0.172
Location × Stimulus Type	0.03	2	36	0.969	0.796	0.002
Anteriority × Location	0.69	1.1	19.76	0.429	0.549	0.037
**Anteriority × Location (Wilk's Λ = 0.55)**	**6.95**	**2**	**17**	**0.006**	-	-
Anteriority × Location × Stimulus Type	0.05	1.49	26.83	0.954	0.745	0.003

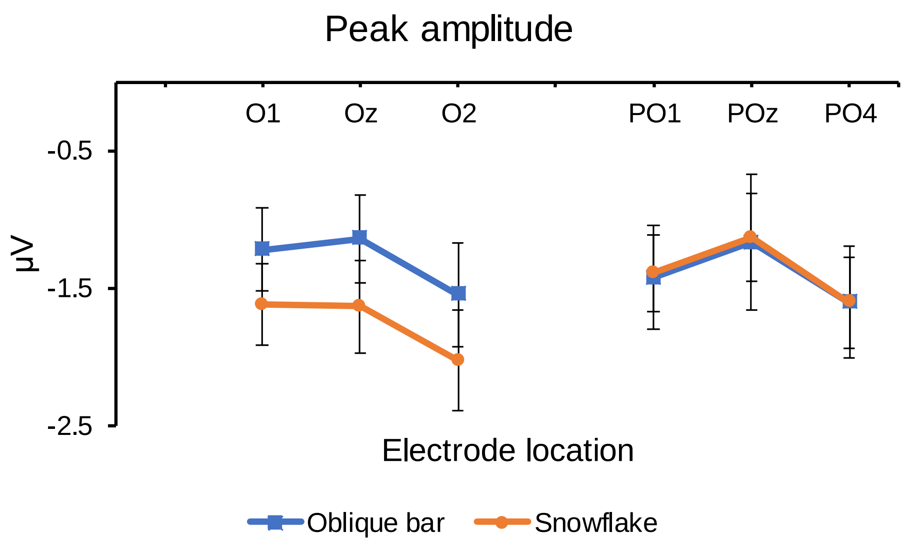

Table S3. Univariate ANOVA for the peak amplitudes at the posterior region for the deviant *minus* control difference wave. Factors: Stimulus Type (oblique bar pattern, snowflake pattern), Location (O1 and PO3, Oz and POz, O2 and PO4), Anteriority (O1, Oz, O2 vs. PO3, POz, PO4). There was no significant difference in peak amplitudes. The main effect for Location ($p = 0.066$) and the Anteriority × Stimulus Type ($p = 0.069$) interaction did not reach significance

deviant *minus* control						
Posterior region (O1, Oz, O2, PO3, POz, PO4)						
Integrated activity						
Effects and interactions	*F*	df 1	df 2	*p*	ε	η_p^2
Stimulus Type	0.78	1	18	0.389	-	0.041
Anteriority	0.69	1	18	0.417	-	0.037
**Location**	**9.31**	**1.53**	**27.5**	**0.002**	**0.764**	**0.341**
**Anteriority × Stimulus Type**	**13.99**	**1**	**18**	**0.002**	**-**	**0.437**
Location × Stimulus Type	1.3	2	36	0.286	0.811	0.067
**Anteriority × Location**	**3.43**	**2**	**36**	**0.043**	**0.851**	**0.16**
Anteriority × Location (Wilk's Λ = 0.788)	2.29	2	17	0.132	-	-
Anteriority × Location × Stimulus Type	2.35	2	36	0.11	0.808	0.115

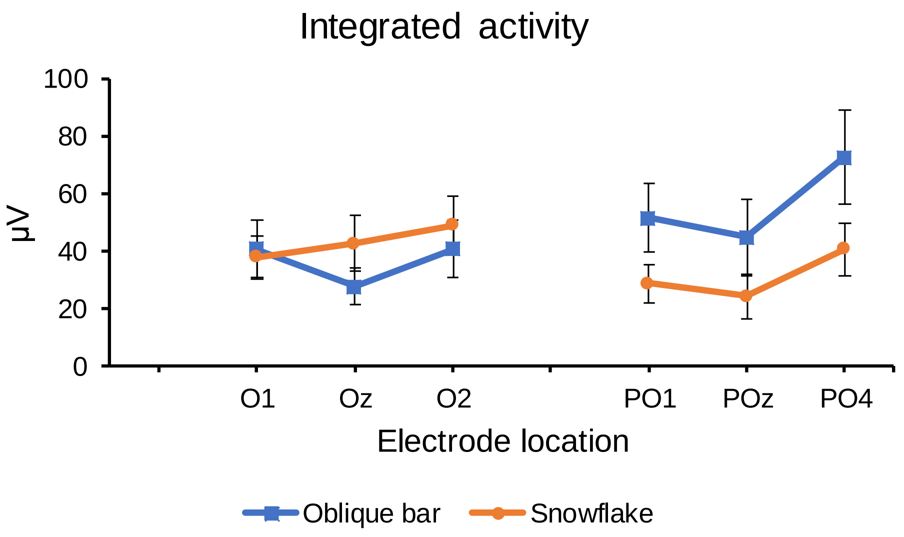

Table S4. Univariate ANOVA for the integrated activity at the posterior region for the deviant *minus* control difference wave. Factors: Stimulus Type (oblique bar pattern, snowflake pattern), Location (O1 and PO3, Oz and POz, O2 and PO4), Anteriority (O1, Oz, O2 vs. PO3, POz, PO4). The integrated activity was larger overall at the O2 and PO4 locations compared to PO3, POz, O1, and Oz (*p*s between 0.0006 and 0.016). It was also larger for the oblique bar deviant at the

PO locations compared to the integrated activity for the snowflake deviant at both O and PO locations ($p = 0.019$ and $p = 0.003$ respectively)

deviant *minus* control						
Frontocentral region (FC1, FCz, FC2, F1, Fz, F2)						
Peak latencies						
Effects and interactions	*F*	df 1	df 2	*p*	ε	η_p^2
Stimulus Type	1.01	1	18	0.329	-	0.053
**Anteriority**	**8.84**	**1**	**18**	**0.008**	**-**	**0.329**
Location	3.66	1.48	26.58	0.051	0.788	0.169
Location (Wilk's Λ = 0.76)	2.28	2	17	0.132	-	-
**Anteriority × Stimulus Type**	**5.69**	**1**	**18**	**0.028**	**-**	**0.240**
Location × Stimulus Type	0.61	1.2	21.76	0.463	0.68	0.033
Anteriority × Location	0.68	1.36	24.46	0.473	0.604	0.036
Anteriority × Location × Stimulus Type	0.11	2	36	0.893	0.825	0.006

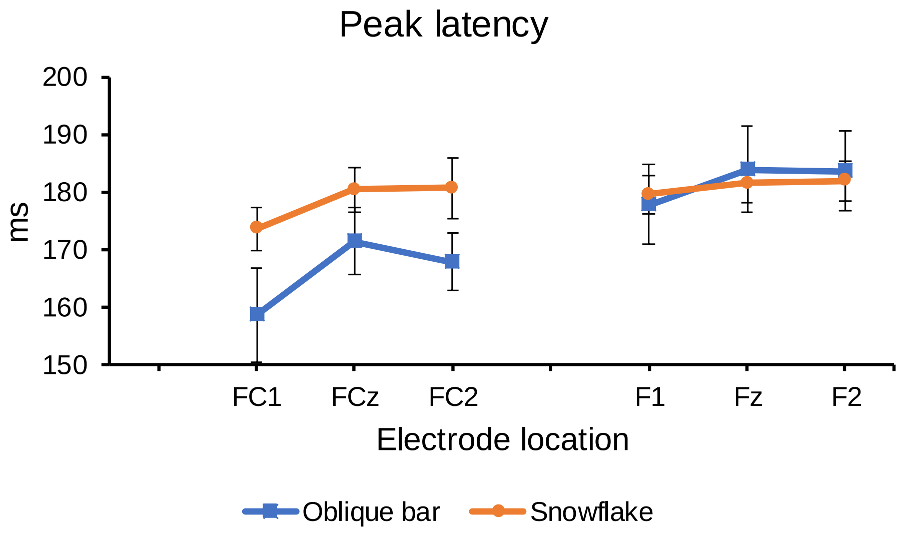

Table S5. Univariate ANOVA for the peak latencies at the anterior region for the deviant *minus* control difference wave. Factors: Stimulus Type (oblique bar pattern, snowflake pattern), Location (FC1 and F1, FCz and Fz, FC2 and F2), Anteriority (FC1, FCz, FC2 vs. F1, Fz, F2). Latency was shorter for the oblique bar pattern as deviant at the FC locations (*p*s between 0.004 and 0.025). The main effect for Location did not reach significance ($p = 0.051$)

deviant *minus* control						
Frontocentral region (FC1, FCz, FC2, F1, Fz, F2)						
Onset latencies						
Effects and interactions	*F*	df 1	df 2	*p*	ε	η_p^2
Stimulus Type	1.99	1	18	0.175	-	-
Anteriority	0.39	1	18	0.543	-	-
Location	0.05	2	36	0.951	0.772	-
Anteriority × Stimulus Type	0.42	1	18	0.528	-	-
Location × Stimulus Type	0.05	2	36	0.956	0.794	-
Anteriority × Location	0.22	2	36	0.805	0.816	-
Anteriority × Location × Stimulus Type	0.24	2	36	0.79	0.802	-

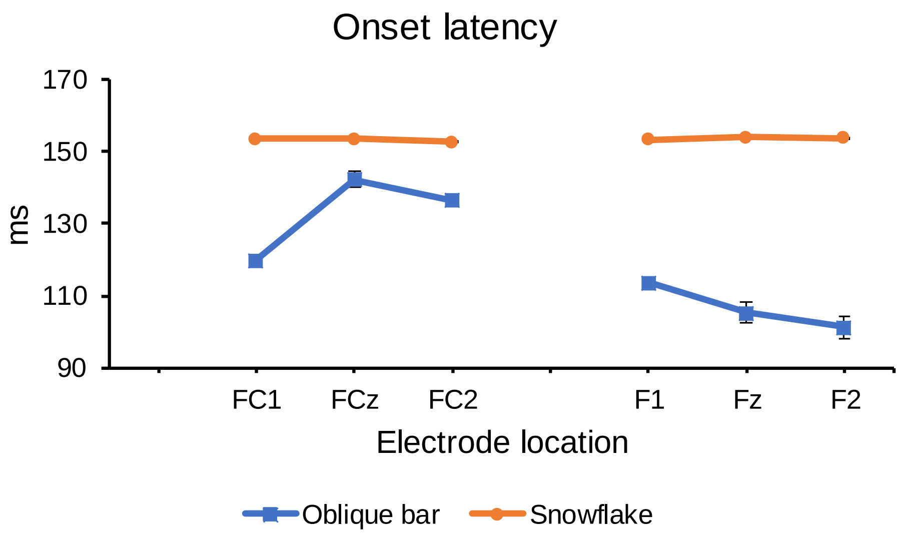

Table S6. Univariate ANOVA for the onset latencies at the anterior region for the deviant *minus* control difference wave. Factors: Stimulus Type (oblique bar pattern, snowflake pattern), Location (FC1 and F1, FCz and Fz, FC2 and F2), Anteriority (FC1, FCz, FC2 vs. F1, Fz, F2). There were no significant differences

deviant *minus* control						
Frontocentral region (FC1, FCz, FC2, F1, Fz, F2)						
Peak amplitude						
Effects and interactions	*F*	df 1	df 2	*p*	ε	η_p^2
Stimulus Type	0.76	1	18	0.393	-	0.04
Anteriority	0.74	1	18	0.4	-	0.04
Location	1.37	1.27	22.88	0.263	0.636	0.07
Anteriority × Stimulus Type	0.03	1	18	0.858	-	0.002
Location × Stimulus Type	0.22	1.35	24.28	0.717	0.674	0.012
Anteriority × Location	0.63	1.21	21.83	0.465	0.606	0.034
Anteriority × Location × Stimulus Type	0.16	1.16	20.96	0.73	0.582	0.009

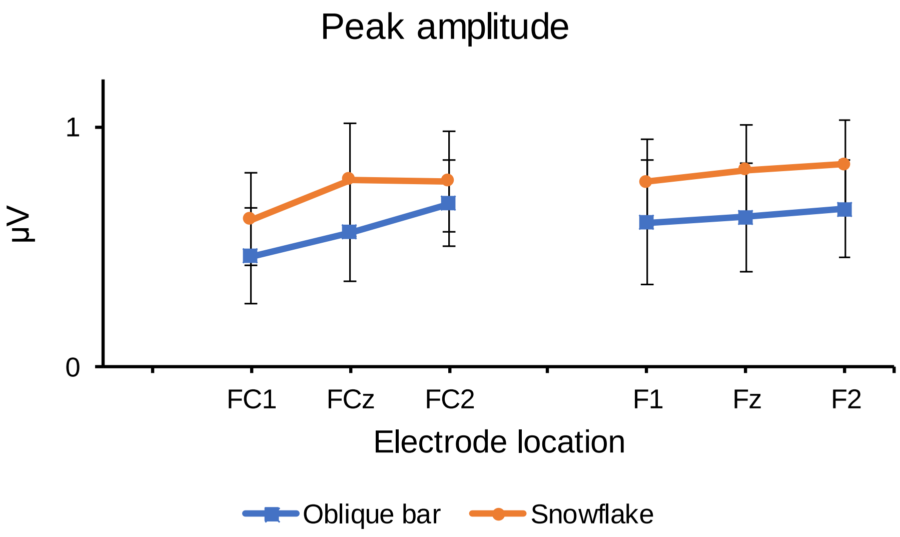

Table S7. Univariate ANOVA for the peak amplitudes at the anterior region for the deviant *minus* control difference wave. Factors: Stimulus Type (oblique bar pattern, snowflake pattern), Location (FC1 and F1, FCz and Fz, FC2 and F2), Anteriority (FC1, FCz, FC2 vs. F1, Fz, F2). There were no significant differences

deviant *minus* control						
Frontocentral region (FC1, FCz, FC2, F1, Fz, F2)						
Integrated activity						
Effects and interactions	*F*	df 1	df 2	*p*	ε	η_p^2
Stimulus Type	0.65	1	18	0.431	-	0.035
**Anteriority**	**10.631**	**1**	**18**	**0.004**	**-**	**0.371**
**Location**	**6.83**	**1.35**	**24.33**	**0.01**	**0.676**	**0.275**
**Anteriority × Stimulus Type**	**5.73**	**1**	**18**	**0.028**	**-**	**0.242**
Location × Stimulus Type	0.51	1.46	26.32	0.551	0.731	0.028
**Anteriority × Location**	**6.14**	**1.23**	**22.05**	**0.017**	**0.612**	**0.254**
Anteriority × Location × Stimulus Type	3.19	1.41	25.3	0.073	0.703	0.150

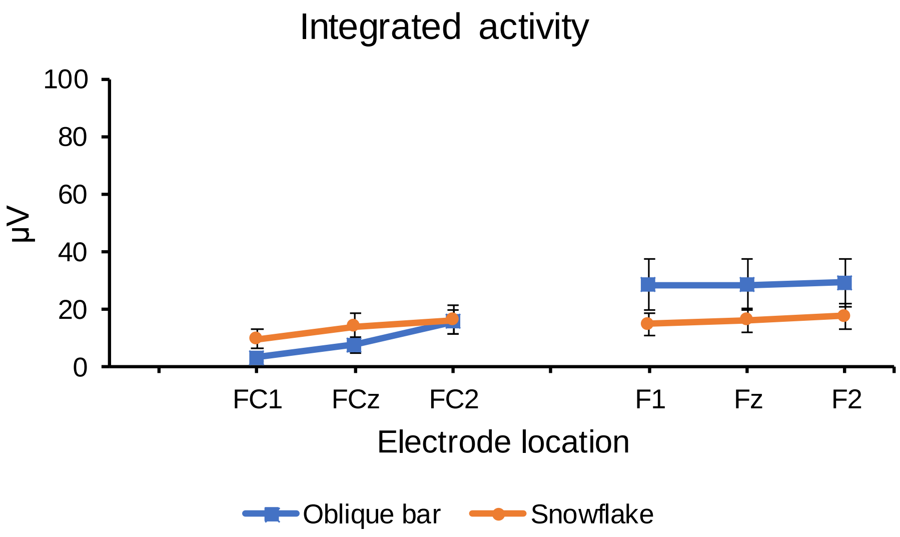

Table S8. Univariate ANOVA for the integrated activity at the anterior region for the deviant *minus* control difference wave. Factors: Stimulus Type (oblique bar pattern, snowflake pattern), Location (FC1 and F1, FCz and Fz, FC2 and F2), Anteriority (FC1, FCz, FC2 vs. F1, Fz, F2). Integrated activity was again larger for the oblique bar deviant (*p*s between 0.005 and 0.097), but only for the F electrode sites. There was an overall increase from FC1 to FC2 for both deviants (*p*s between 0.0002 and 0.088). The three-way interaction did not reach significance (p = 0.073)

control *minus* standard						
Posterior region (O1, Oz, O2, PO3, POz, PO4)						
Peak latencies						
Effects and interactions	*F*	df 1	df 2	*p*	ε	η_p^2
**Stimulus Type**	**238.69**	**1**	**18**	**<0.001**	**-**	**0.93**
**Anteriority**	**5.83**	**1**	**18**	**<0.001**	**-**	**0.468**
**Location**	**7.64**	**2**	**36**	**0.002**	**0.995**	**0.298**
**Anteriority × Stimulus Type**	**12.3**	**1**	**18**	**0.003**	**-**	**0.406**
Location × Stimulus Type	3.21	2	36	0.052	0.932	0.151
Location × Stimulus Type (Wilk's Λ = 0.78)	2.39	2	17	0.122	-	-
**Anteriority × Location**	**4.13**	**1.47**	**26.47**	**0.038**	**0.735**	**0.187**
Anteriority × Location (Wilk's Λ = 0.767)	2.584	2	17	0.105	-	-
Anteriority × Location × Stimulus Type	2.3	1.52	27.35	0.13	0.76	0.113

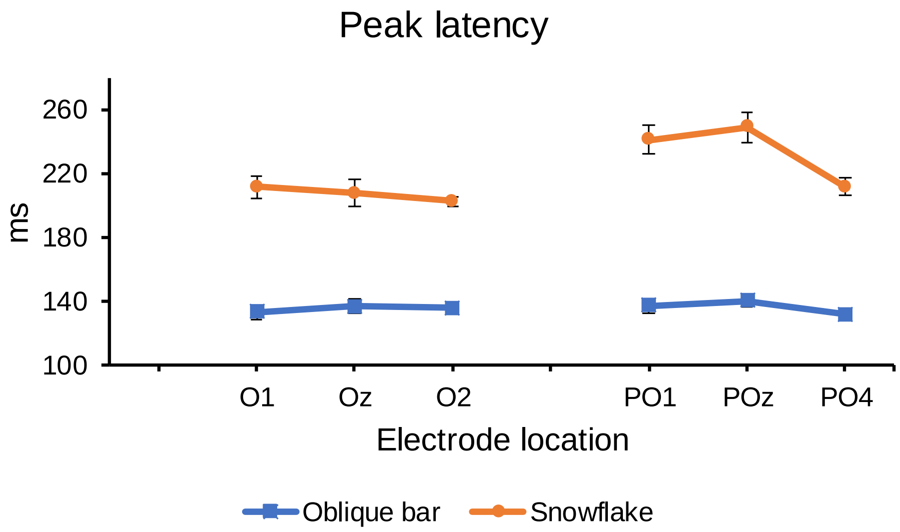

Table S9. Univariate ANOVA for the peak latencies at the posterior region for control *minus* standard difference wave. Factors: Stimulus Type (oblique bar pattern, snowflake pattern), Location (O1 and PO3, Oz and POz, O2 and PO4), Anteriority (O1, Oz, O2 vs. PO3, POz, PO2). The peak latency for the snowflake pattern as standard was overall longer at the posterior region,

and longer at the PO sites than at the O sites (ps < 0.001). The latencies are overall longer at PO3 and POz than all other sites (ps between 0.0003 and 0.021). The Location × Stimulus Type interaction did not reach significance ($p = 0.052$)

control *minus* standard						
Posterior region (O1, Oz, O2, PO3, POz, PO4)						
Onset latencies						
Effects and interactions	*F*	df 1	df 2	*p*	ε	η_p^2
**Stimulus Type**	**121.2**	**1**	**18**	**<0.001**	**-**	**-**
**Anteriority**	**7.13**	**1**	**18**	**0.016**	**-**	**-**
Location	0.74	2	36	0.483	0.787	-
Anteriority × Stimulus Type	0.72	1	18	0.409	-	-
Location × Stimulus Type	3.04	2	36	0.06	0.848	-
Location × Stimulus Type (Wilk's Λ = 0.011)	2.36	2	17	0.125	-	-
**Anteriority × Location**	**3.51**	**2**	**36**	**0.041**	**0.795**	**-**
Anteriority × Location (Wilk's Λ = 0.012	2.2	2	17	0.141	-	-
Anteriority × Location × Stimulus Type	0.37	2	36	0.691	0.877	-

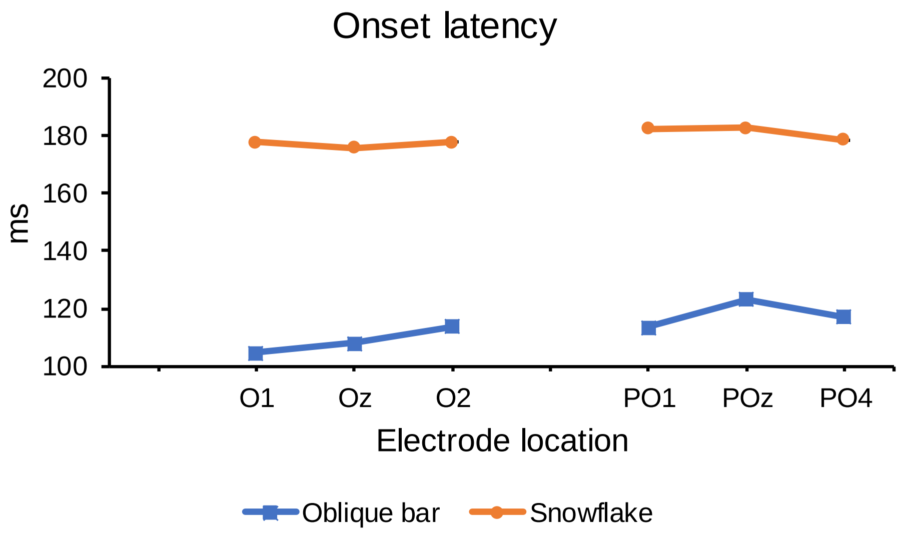

Table S10. Univariate ANOVA for the onset latencies at the posterior region for control *minus* standard difference wave. Factors: Stimulus Type (oblique bar pattern, snowflake pattern), Location (O1 and PO3, Oz and POz, O2 and PO4), Anteriority (O1, Oz, O2 vs. PO3, POz, PO2). Overall the onset latencies are longer at the F sites than at the FC sites ($p < 0.001$). Onset

latencies were also longer for the snowflake pattern as standard ($p < 0.001$). The Location × Stimulus Type interaction did not reach significance (p=0.06)

control *minus* standard						
Posterior region (O1, Oz, O2, PO3, POz, PO4)						
Peak amplitudes						
Effects and interactions	*F*	df 1	df 2	*p*	ε	η_p^2
**Stimulus Type**	**4.46**	**1**	**18**	**0.049**	**-**	**0.199**
**Anteriority**	**12.32**	**1**	**18**	**0.003**	**-**	**0.406**
Location	1.48	1.27	22.8	0.243	0.633	0.076
**Location (Wilk's Λ = 0.673)**	**4.12**	**2**	**17**	**0.035**	**0.633**	-
**Anteriority × Stimulus Type**	**10.95**	**1**	**18**	**0.004**	**-**	**0.378**
Location × Stimulus Type	1.79	2	36	0.181	0.773	0.091
**Anteriority × Location**	**5.71**	**2**	**36**	**0.007**	**0.832**	**0.241**
Anteriority × Location × Stimulus Type	0.98	2	36	0.907	0.793	0.005

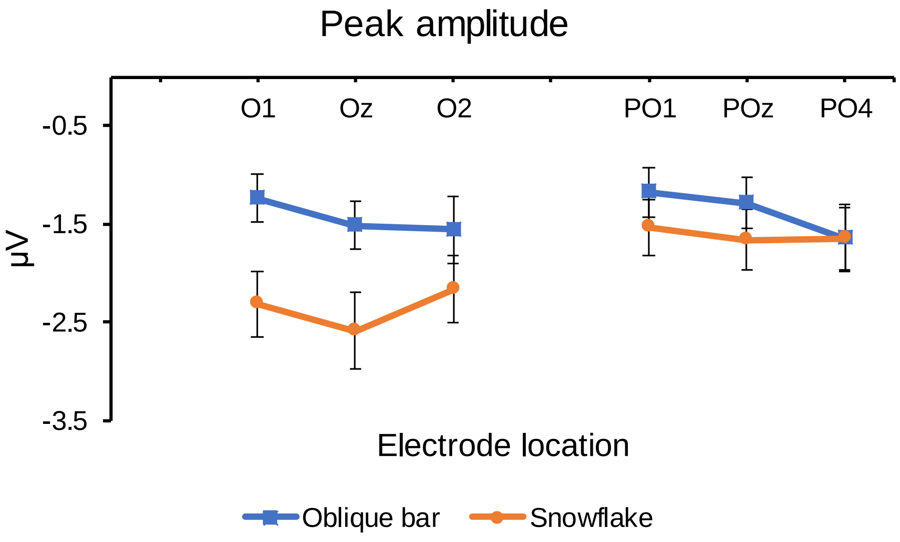

Table S11. Univariate ANOVA for the peak amplitudes at the posterior region for control *minus* standard difference wave. Factors: Stimulus Type (oblique bar pattern, snowflake pattern), Location (O1 and PO3, Oz and POz, O2 and PO4), Anteriority (O1, Oz, O2 vs. PO3, POz, PO2). The peak amplitude was larger for the snowflake standard at O sites ($p < 0.001$). Overall peak amplitudes were larger at Oz compared to O1 ($p = 0.01$), and larger at PO4 compared to PO3 ($p =$

0.006). Peak amplitudes at O sites were larger than the peak amplitudes at PO3 and POz (*p*s < 0.004)

control *minus* standard						
Posterior region (O1, Oz, O2, PO3, POz, PO4)						
Integrated activity						
Effects and interactions	*F*	df 1	df 2	*p*	ε	η_p^2
**Stimulus Type**	**10.57**	**1**	**18**	**0.004**	**-**	**0.37**
**Anteriority**	**23.12**	**1**	**18**	**<0.001**	**-**	**0.562**
Location	2.45	2	36	0.101	0.82	0.12
**Location (Wilk's Λ = 0.673)**	**4.13**	**2**	**17**	**0.035**		-
**Anteriority × Stimulus Type**	**9.47**	**1**	**18**	**0.007**	**-**	**0.345**
Location × Stimulus Type	0.05	2	36	0.951	0.958	0.003
Anteriority × Location	2.24	2	36	0.121	0.852	0.111
Anteriority × Location × Stimulus Type	2.08	2	36	0.139	0.89	0.104

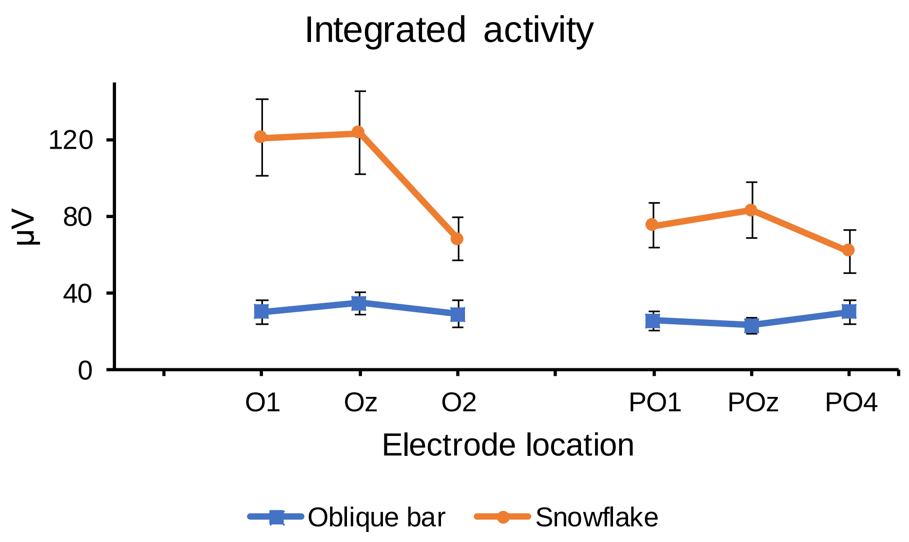

Table S12. Univariate ANOVA for the peak latencies at the posterior region for control *minus* standard difference wave. Factors: Stimulus Type (oblique bar pattern, snowflake pattern), Location (O1 and PO3, Oz and POz, O2 and PO4), Anteriority (O1, Oz, O2 vs. PO3, POz, PO2). The integrated activity for the snowflake pattern as standard was significantly larger at the O sites compared to the integrated activity for the oblique bar pattern as standard at both PO and O sites

as well as larger than the integrated activity for the snowflake pattern as standard at the PO sites ($ps < 0.001$). The latter tended to be larger than the integrated activity for the oblique bar pattern as standard at the PO sites ($p = 0.06$)

control *minus* standard						
Frontocentral region (FC1, FCz, FC2, F1, Fz, F2)						
Peak latencies						
Effects and interactions	*F*	df 1	df 2	*p*	ε	η_p^2
**Stimulus Type**	**166.92**	**1**	**18**	**<0.001**	**-**	**0.903**
Anteriority	4.37	1	18	0.051	-	0.195
Location	1.53	2	36	0.23	0.999	0.079
Anteriority × Stimulus Type	1.89	1	18	0.186	-	0.095
Location × Stimulus Type	0.18	1.46	26.22	0.77	0.728	0.01
Anteriority × Location	1.77	2	36	0.185	0.985	0.089
Anteriority × Location × Stimulus Type	0.76	2	36	0.475	0.853	0.041

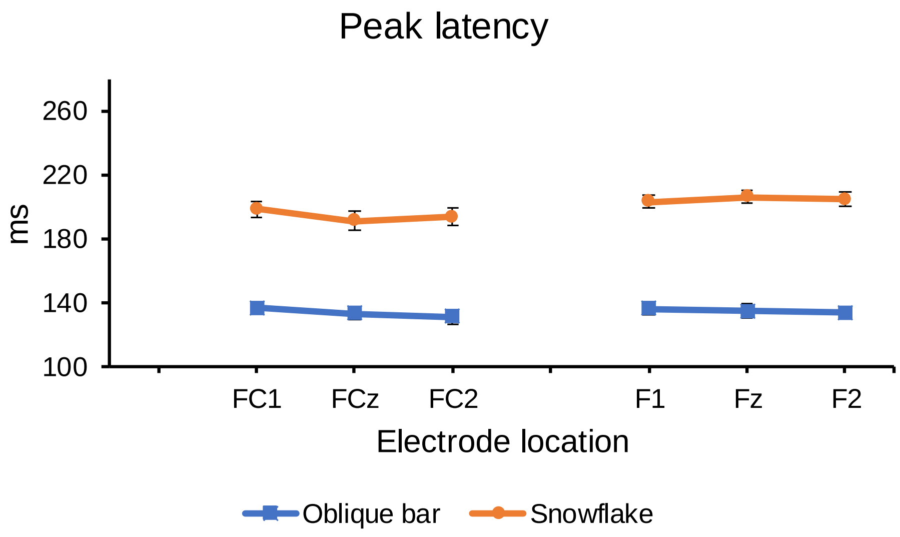

Table S13. Univariate ANOVA for the peak latencies at the anterior region for control *minus* standard difference wave. Factors: Stimulus Type (oblique bar pattern, snowflake pattern), Location (FC1 and F1, FCz and Fz, FC2 and F2), Anteriority (FC1, FCz, FC2 vs. F1, Fz, F2). There was a trend for a shorter latency at the F sites. Overall the latency to the snowflake pattern as standard was longer. The main effect of Anteriority did not reach significance (p = 0.051)

control *minus* standard						
Frontocentral region (FC1, FCz, FC2, F1, Fz, F2)						
Onset latencies						
Effects and interactions	*F*	df 1	df 2	*p*	ε	η_p^2
**Stimulus Type**	**6.57**	**1**	**18**	**0.02**	**-**	**-**
Anteriority	0.22	1	18	0.645	-	-
Location	0.14	2	36	0.874	0.846	-
Anteriority × Stimulus Type	0.73	1	18	0.79	-	-
Location × Stimulus Type	0.03	2	36	0.971	0.751	-
Anteriority × Location	0.04	2	36	0.959	0.852	-
Anteriority × Location × Stimulus Type	0.3	2	36	0.973	0.829	-

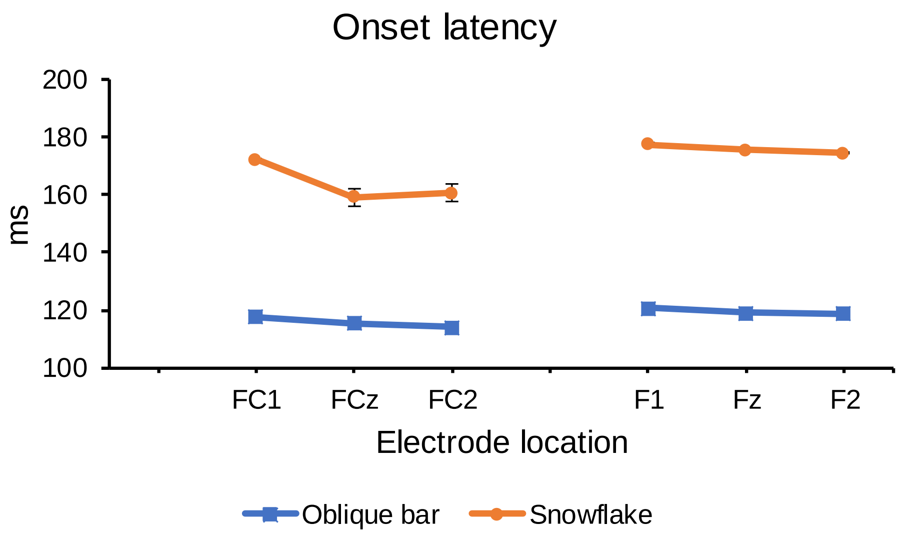

Table S14. Univariate ANOVA for the onset latencies at the anterior region for control *minus* standard difference wave. Factors: Stimulus Type (oblique bar pattern, snowflake pattern), Location (FC1 and F1, FCz and Fz, FC2 and F2), Anteriority (FC1, FCz, FC2 vs. F1, Fz, F2). The onset latency for the snowflake pattern as standard was longer than that for the oblique bar pattern as standard

control *minus* standard						
Frontocentral region (FC1, FCz, FC2, F1, Fz, F2)						
Peak amplitudes						
Effects and interactions	*F*	df 1	df 2	*p*	ε	η_p^2
Stimulus Type	1.31	1	18	0.268	-	0.068
Anteriority	1.38	1	18	0.256	-	0.071
Location	1.03	1.32	23.79	0.342	0.661	0.054
Anteriority × Stimulus Type	1.08	1	18	0.313	-	0.056
Location × Stimulus Type	0.01	2	36	0.993	0.824	0.000
Anteriority × Location	2.73	2	36	0.079	0.943	0.132
Anteriority × Location × Stimulus Type	0.13	2	36	0.883	0.944	0.007

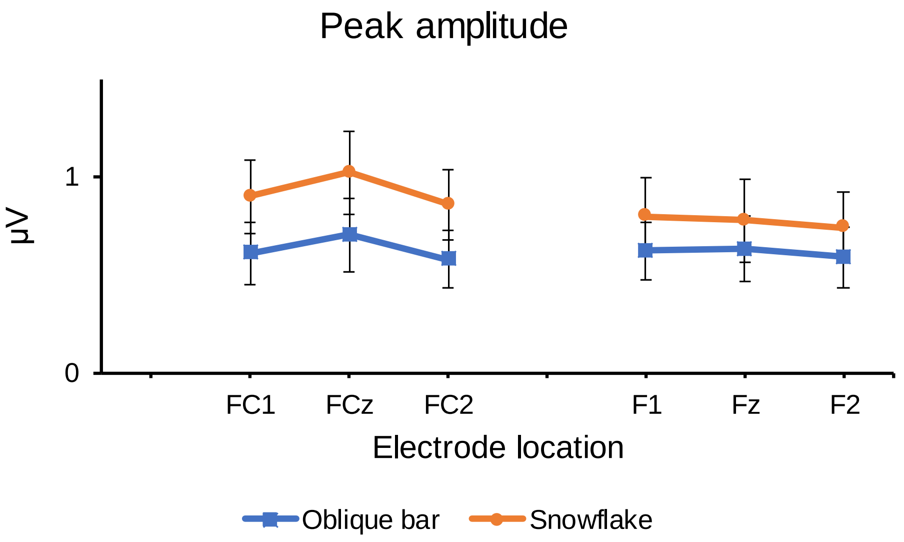

Table S15. Univariate ANOVA for the peak amplitudes at the anterior region for control *minus* standard difference wave. Factors: Stimulus Type (oblique bar pattern, snowflake pattern), Location (FC1 and F1, FCz and Fz, FC2 and F2), Anteriority (FC1, FCz, FC2 vs. F1, Fz, F2). The Anteriority × Location interaction did not reach significance (p = 0.079)

control *minus* standard						
Frontocentral region (FC1, FCz, FC2, F1, Fz, F2)						
Integrated activity						
Effects and interactions	*F*	df 1	df 2	*p*	ε	η_p^2
**Stimulus Type**	**10.75**	**1**	**18**	**0.004**	**-**	**0.374**
**Anteriority**	**11.45**	**1**	**18**	**0.003**	**-**	**0.389**
**Location**	**5.31**	**2**	**36**	**0.01**	**0.85**	**0.228**
**Anteriority × Stimulus Type**	**7.25**	**1**	**18**	**0.015**	**-**	**0.287**
Location × Stimulus Type	1.46	2	36	0.245	0.869	0.075
**Anteriority × Location**	**12.34**	**2**	**36**	**<0.001**	**0.932**	**0.407**
**Anteriority × Location × Stimulus Type**	**5.25**	**1.33**	**23.86**	**0.023**	**0.663**	**0.226**

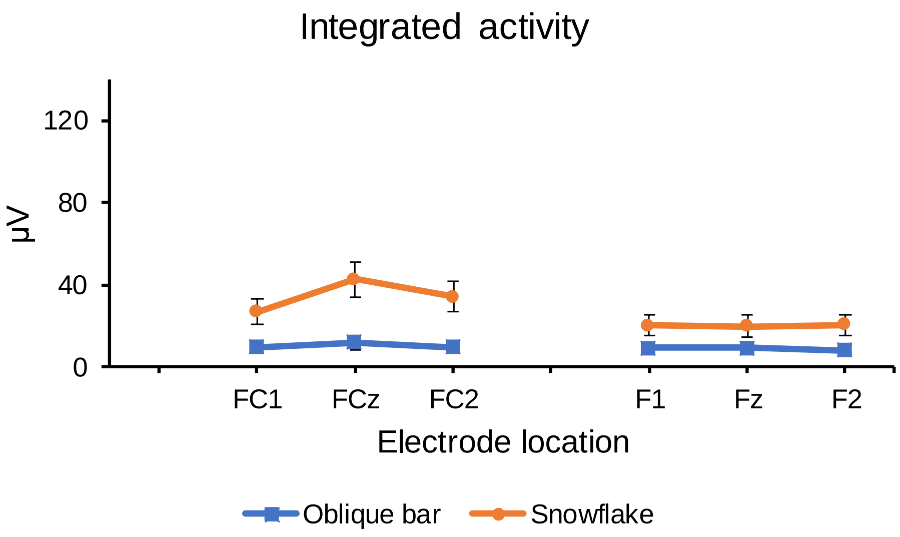

Table S16. Univariate ANOVA for the integrated activity at the anterior region for control *minus* standard difference wave. Factors: Stimulus Type (oblique bar pattern, snowflake pattern), Location (FC1 and F1, FCz and Fz, FC2 and F2), Anteriority (FC1, FCz, FC2 vs. F1, Fz, F2). The integrated activity for the snowflake pattern was larger at FCz than at FC1 and FC2 (*p*s < 0.011), and smaller at all F sites that at FCz and F2 (*p*s < 0.001). The integrated activity for the snowflake pattern was larger overall than the integrated activity for the oblique bar (*p* = 0.004)
