# Supplementary material for: Visual mismatch negativity and stimulus-specific adaptation: the role of stimulus complexity
Source: Exp Brain Res. 2019 Feb 26;237(5):1179–94. doi: 10.1007/s00221-019-05494-2 (PMC6557884; doi:10.1007/s00221-019-05494-2)
Supplement: Supplementary file 2 — Supplementary material 2. ERPs to the stimuli in the two equiprobable condition (PDF 371 KB) [file 221_2019_5494_MOESM2_ESM.pdf]

Online Resource 2

Article title: Visual mismatch negativity and stimulus-specific adaptation: The role of stimulus complexity

Journal: Experimental Brain Research

Authors:

Petia Kojouharova, Institute of Cognitive Neuroscience and Psychology, Research Centre for Natural Sciences, Hungarian Academy of Sciences; Doctoral School of Psychology, Eötvös Loránd University. kojouharova.petia@ttk.mta.hu

Domonkos File, Doctoral School of Psychology, Eötvös Loránd University; Institute of Psychology, Eötvös Loránd University; Institute of Cognitive Neuroscience and Psychology, Research Centre for Natural Sciences, Hungarian Academy of Sciences

István Sulykos, Institute of Cognitive Neuroscience and Psychology, Research Centre for Natural Sciences, Hungarian Academy of Sciences.

István Czigler, Institute of Cognitive Neuroscience and Psychology, Research Centre for Natural Sciences, Hungarian Academy of Sciences.

Supplementary Information 2

The ERPs to the stimuli in the equiprobable conditions are shown on Figure S1. The ERPs to the oblique bar patterns are fairly similar. The ERPs to the complex shapes, though similar in morphology, differ from each other, but not in a discernible systematic way.

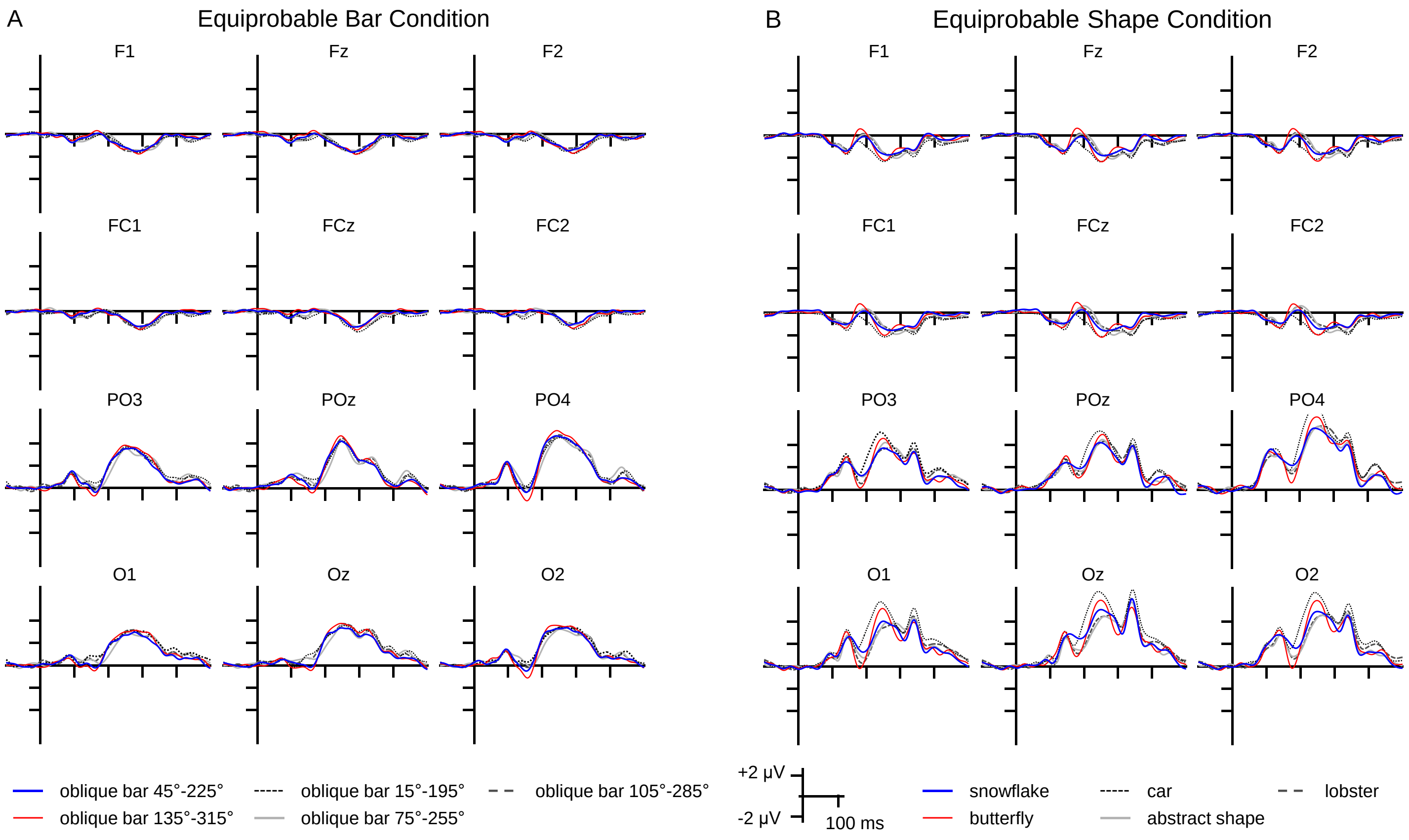

Fig S1. ERPs to the stimuli in the Equiprobable Bar Condition (A) and the Equiprobable Shape Condition (B)
