# Supplementary material for: Visual mismatch negativity and stimulus-specific adaptation: the role of stimulus complexity
Source: Exp Brain Res. 2019 Feb 26;237(5):1179–94. doi: 10.1007/s00221-019-05494-2 (PMC6557884; doi:10.1007/s00221-019-05494-2)
Supplement: Supplementary file 3 — Supplementary material 3. Adaptation of the standard stimuli in the Deviant Snowflake Oddball condition and in the Deviant Bar Oddball condition (PDF 398 KB) [file 221_2019_5494_MOESM3_ESM.pdf]

Online Resource 3

Article title: Visual mismatch negativity and stimulus-specific adaptation: The role of stimulus complexity

Journal: Experimental Brain Research

Authors:

Petia Kojouharova, Institute of Cognitive Neuroscience and Psychology, Research Centre for Natural Sciences, Hungarian Academy of Sciences; Doctoral School of Psychology, Eötvös Loránd University. kojouharova.petia@ttk.mta.hu

Domonkos File, Doctoral School of Psychology, Eötvös Loránd University; Institute of Psychology, Eötvös Loránd University; Institute of Cognitive Neuroscience and Psychology, Research Centre for Natural Sciences, Hungarian Academy of Sciences

István Sulykos, Institute of Cognitive Neuroscience and Psychology, Research Centre for Natural Sciences, Hungarian Academy of Sciences.

István Czigler, Institute of Cognitive Neuroscience and Psychology, Research Centre for Natural Sciences, Hungarian Academy of Sciences.

Supplementary Information 3

One unexpected finding of our study was the large adaptation difference for the standard stimuli between the two oddball conditions. One additional question is whether the adaptation occurs gradually or relatively fast for the two types of standard stimulus.

We examined the time course of the adaptation to the standard stimuli for each oddball condition by dividing the standard stimuli in each oddball sequence into four equal groups of 40 stimuli (Standard 1, Standard 2, Standard 3, and Standard 4). This was carried out by removing all 40 deviant stimuli from each sequence (each sequence consisted of 200 stimuli) and dividing the remaining 160 standard stimuli into four groups following the order in which they appeared in the sequence, e.g., the Standard 1 group was comprised of the first 40 standard stimuli of the sequence. Because each condition had three sequences (see the Stimuli and Procedure in the main text), the final number of standard stimuli in each group was 120. After rejecting the epochs with an amplitude change exceeding 100 μV on any channel (for further details see the Data Analysis section), the remaining epochs were averaged for each group across the sequences, resulting in an average of 111-113 epochs per participant per group. The averaged ERPs for all groups and for both standard stimulus types (oblique bar patterns and snowflake patterns) are depicted on Figure S1 for the electrode sites in both the posterior (O1, Oz, O2, PO3, POz, PO4) and the frontocentral (FC1, FCz, FC2, F1, Fz, F2) region analyzed in the main study. For both the oblique bar pattern and the snowflake pattern the ERP to Standard 1 is markedly different from the ERPs to Standard 2, Standard 3, and Standard 4, whereas the ERPs to the latter three groups look similar.

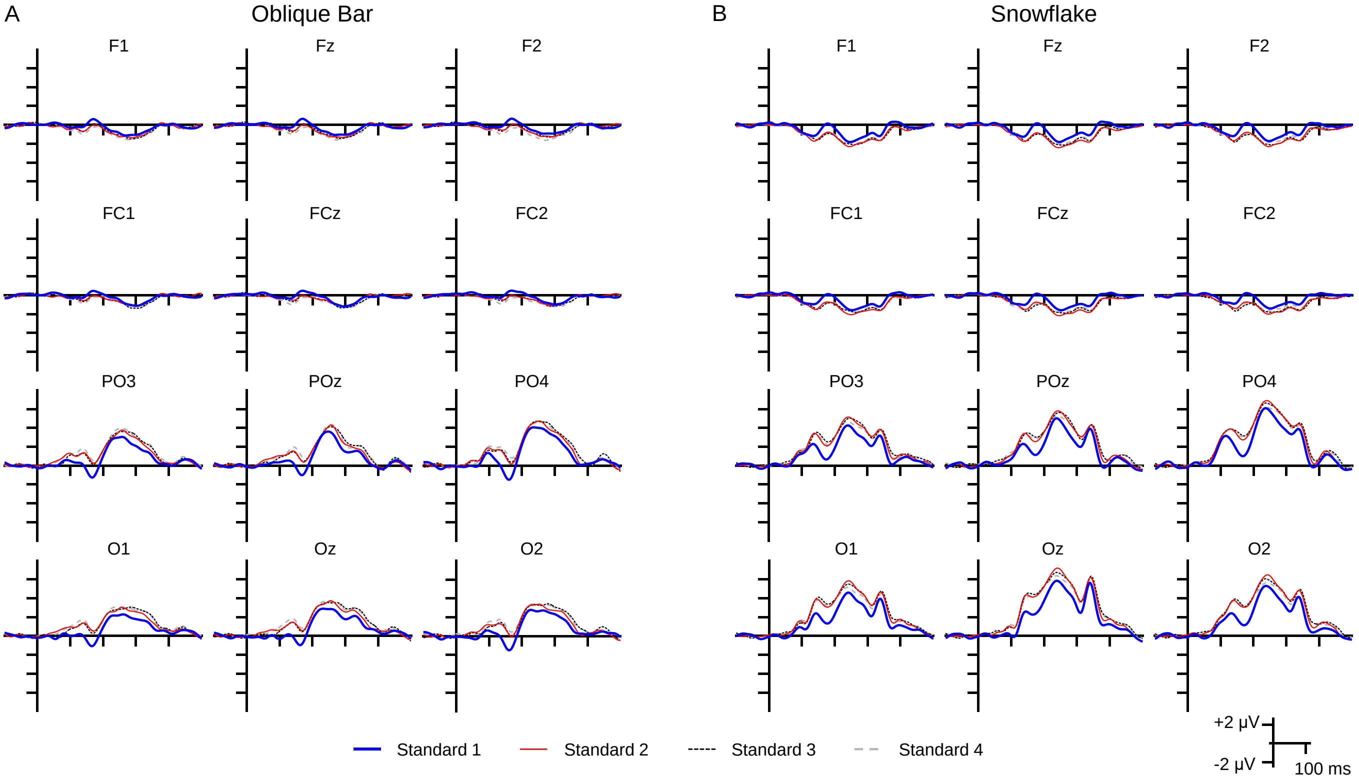

Fig S1. ERPs to Standard 1, Standard 2, Standard 3, and Standard 4 for the oblique bar pattern (A) and the snowflake pattern (B) registered at the posterior and at the frontocentral region

All possible difference potentials were calculated (e.g., Standard 1 *minus* Standard 2, Standard 1 *minus* Standard 3, Standard 2 *minus* Standard 4, and so forth). Then point-by-point one-sample t-tests were run on the difference potentials to identify consecutive data points for which the difference deviates significantly from zero in either direction. Figure S2 illustrates the difference between Standard 1 and Standard 2 (Standard 1 *minus* Standard 2) for the oblique bar pattern as standard and for the snowflake pattern as standard, including the ranges of consecutive significant deviations. The results for the Standard 1 *minus* Standard 3 and Standard 1 *minus* Standard 4 difference potentials were very similar, whereas for the remaining difference potentials such difference ranges did not emerge.

In brief, the standard stimuli are quickly adapted in the beginning of the sequence for both simple and complex stimuli.

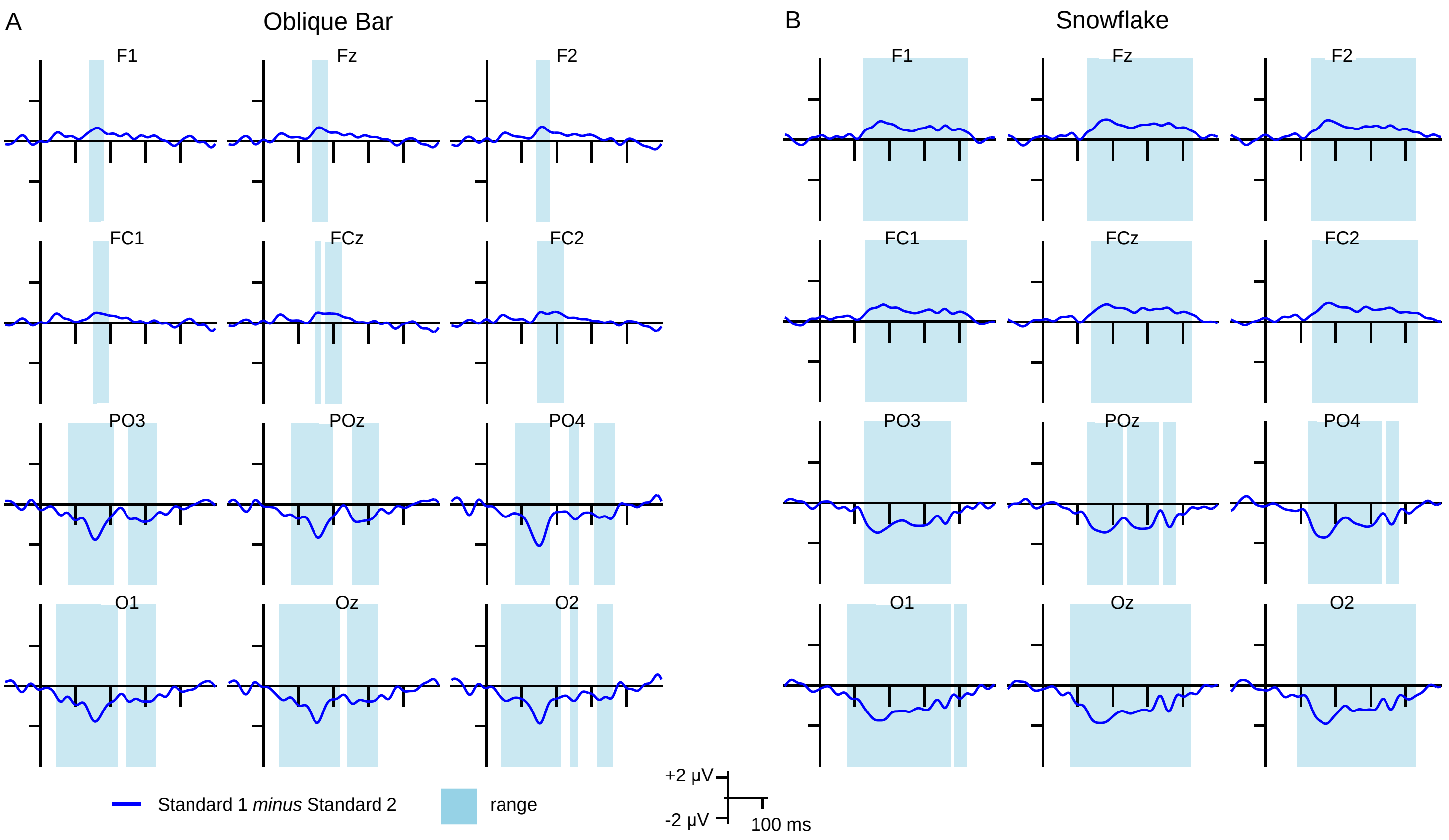

Fig S2. The Standard 1 *minus* Standard 2 difference potentials for the oblique bar pattern (A) and the snowflake pattern (B) as standard stimuli registered at the posterior and at the frontocentral region
