# Supplementary material for: Visual mismatch negativity and stimulus-specific adaptation: the role of stimulus complexity
Source: Exp Brain Res. 2019 Feb 26;237(5):1179–94. doi: 10.1007/s00221-019-05494-2 (PMC6557884; doi:10.1007/s00221-019-05494-2)
Supplement: Supplementary file 4 — Supplementary material 4. A comparison of topographic maps with the permutation method described by Karniski et al. (1994) (PDF 954 KB) [file 221_2019_5494_MOESM4_ESM.pdf]

Online Resource 4

Article title: Visual mismatch negativity and stimulus-specific adaptation: The role of stimulus complexity

Journal: Experimental Brain Research

Authors:

Petia Kojouharova, Institute of Cognitive Neuroscience and Psychology, Research Centre for Natural Sciences, Hungarian Academy of Sciences; Doctoral School of Psychology, Eötvös Loránd University. kojouharova.petia@ttk.mta.hu

Domonkos File, Doctoral School of Psychology, Eötvös Loránd University; Institute of Psychology, Eötvös Loránd University; Institute of Cognitive Neuroscience and Psychology, Research Centre for Natural Sciences, Hungarian Academy of Sciences

István Sulykos, Institute of Cognitive Neuroscience and Psychology, Research Centre for Natural Sciences, Hungarian Academy of Sciences.

István Czigler, Institute of Cognitive Neuroscience and Psychology, Research Centre for Natural Sciences, Hungarian Academy of Sciences.

Supplementary Information 4

Comparison of topographic maps

Tables S1 an S2 show the topographic maps for the 100-300 ms period (divided into periods of 20 ms) for both Stimulus Types for the deviant *minus* control and the control *minus* standard differences. For each period the scalp distributions were compared with the permutation method described by Karniski, Blair, and Snider (1994), and the *p* value of the comparison is displayed. In this method a distribution of the sums of the t-test comparisons (multiple hypothesis testing) for all possible permutations of the topographic maps is created, and the original comparison is evaluated by how extreme it is in that distribution. The results suggest that the deviant *minus* control difference starts earlier for the oblique bar pattern as deviant and lasts somewhat longer, but the scalp distributions do not differ from each other (compared in the 160-178 ms time window) which may indicate the same source for the visual mismatch negativity. The control *minus* standard difference is early and short for the oblique bar pattern as standard and later and somewhat longer for the snowflake pattern as standard. In the latter case the scalp distribution of the oblique bar pattern as a standard in the 140-158 ms time window was compared to the scalp distribution of the snowflake pattern as standard in the 200-218 ms time window. The difference did not reach significance, $p = 0.0868$, but may still indicate a difference between the two conditions concerning stimulus-specific adaptation.

Karniski W, Blair RC, & Snider AD (1994) An exact statistical method for comparing topographic maps, with any number of subjects and electrodes. Brain Topogr 6:203-210.

deviant *minus* control										
Time window	100-118 ms	120-138 ms	140-158 ms	160-178 ms	180-198 ms	200-218 ms	220-238 ms	240-258 ms	260-278 ms	280-298 ms
oblique bar deviant	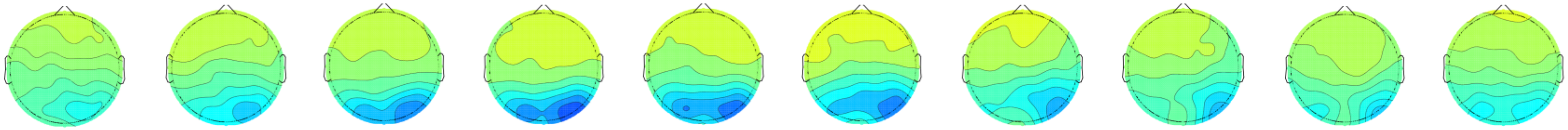									
snowflake deviant	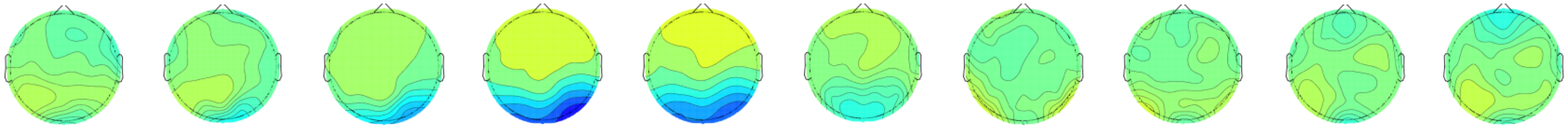									
p value	0.042	0.022	0.278	0.831	0.823	0.129	0.043	0.058	0.128	0.011

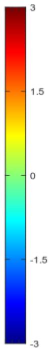

Table S1. Results from the permutation tests and topographic maps for the deviant *minus* control difference

control *minus* standard										
Time window	100-118 ms	120-138 ms	140-158 ms	160-178 ms	180-198 ms	200-218 ms	220-238 ms	240-258 ms	260-278 ms	280-298 ms
oblique bar standard	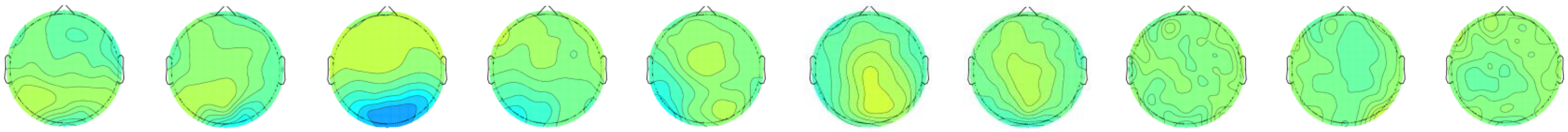									
snowflake standard	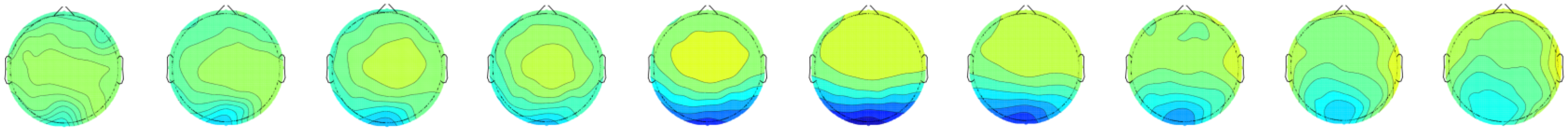									
p value	0.818	0.017	0.007	0.111	0.011	<0.001	0.003	0.048	0.092	0.24

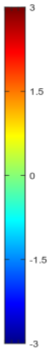

Table S2. Results from the permutation tests and topographic maps for the control *minus* standard difference
